# Maternal grandmothers buffer the effects of ethnic discrimination among pregnant Latina mothers

**DOI:** 10.1017/ehs.2023.27

**Published:** 2023-11-09

**Authors:** Delaney A. Knorr, Molly M. Fox

**Affiliations:** 1 Department of Anthropology, University of California, Los Angeles, CA 90095, USA; 2 Department of Psychiatry & Biobehavioral Sciences, University of California Los Angeles, Los Angeles, CA 90095, USA

**Keywords:** allomothers, grandmothers, discrimination, emotional support, prenatal psychological distress

## Abstract

Ethnic discrimination during pregnancy is linked to maternal psychological distress, adverse birth outcomes and increased offspring morbidity and mortality. An evolutionary perspective reframes offspring health issues as a risk to maternal fitness. We argue that kin may be evolutionarily motivated to buffer psychosocial stressors for the mother during pregnancy. Previously, we found that the relationship of a pregnant woman with her own mother (fetus’ maternal grandmother) had a positive association on maternal prenatal psychology, above and beyond her relationship with her fetus’ father. Here, we ask if grandmothers buffer mothers’ prenatal psychological distress from ethnic discrimination. Using self-report data collected from 216 pregnant Latina women living in Southern California, we found discrimination to be significantly, positively associated with depression, anxiety, and stress in linear regression models. Maternal grandmother communication attenuated the association of discrimination and all three psychological distress measures, adjusting for the mother's relationship with the father. Maternal grandmother emotional support similarly significantly moderated the relationship of discrimination with depression and anxiety. We did not observe any significant interactions for paternal grandmother relationships. Geographic proximity was not a significant stress buffer. Results suggest the important role maternal grandmothers play in perinatal mental health, and that these benefits exist uncoupled from geographic proximity.

## Graphical abstract

**Figure:**
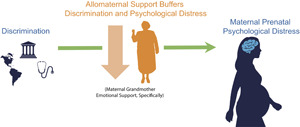

**Social media summary:** Grandmother–mother relationships buffer against ill-effects of ethnic discrimination on prenatal psychological distress.

## 1. Introduction

The framework of developmental origins of health and disease, DOHaD, describes how mothers’ prenatal environmental exposures (including psychosocial stressors) can create long-term phenotypic changes in offspring that lead to elevated disease risk across the life course (Gluckman et al., 2008). When pregnant women experience psychosocial stressors, perturbations to fetal development, birth timing and infant development can occur. Prenatal maternal psychological distress (including stress, anxiety, and depression) has also been associated with low-birth weight and preterm birth (Grigoriadis et al., 2013, 2018; Grote et al., 2010), which in turn are associated with the offspring's long-term disease-risk, including cardiovascular disease, obesity, diabetes and psychopathology (Callaghan et al., 2006; Eshete et al., 2019).

**Figure 1.**
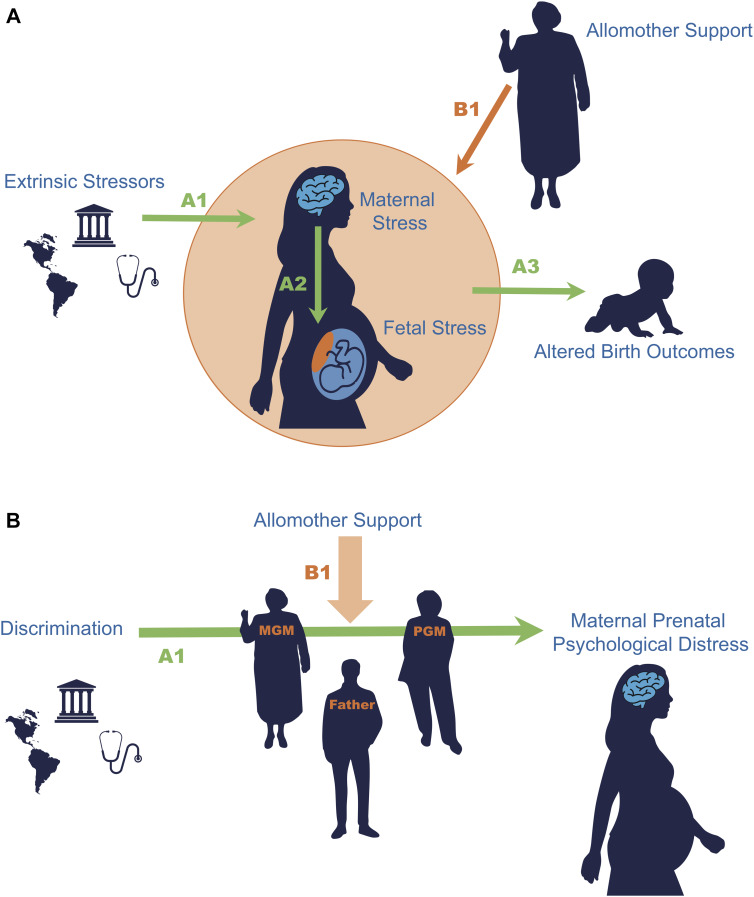
Panel A depicts a broader conceptual model connected to the overall state of the literature. There is an epidemiological trend of extrinsic stressors relating to altered birth outcomes (path A). This trend likely stems from stressors negatively influencing maternal psychological and physiological stress systems (path A1), which are biologically signaled through the placenta in a way that impacts fetal growth and development trajectories (path A2) that ultimately lead to altered birth outcomes like low birth weight (path A3). Overall, we suggest that allomothers may be motivated to buffer this cascade (path B1) for the benefit of the developing infant. Panel B depicts the conceptual model of this study. In this paper, we focus on how allomaternal relationship characteristics (particularly between grandmothers and mothers) buffers (B1) the relationship of extrinsic stressors and maternal distress (A1). This particular paper focuses on how the relationship of discrimination and psychological distress is moderated by allomother relationship characteristics. The abbreviations MGM and PGM stand for maternal and paternal grandmother, respectively.

We have argued elsewhere that allomothers may be adaptively motivated to offset prenatal risks to the offspring for inclusive fitness benefits, extending allomaternal care to the prenatal period (Figure 1A; Knorr & Fox, 2023). Thus, allomothers may buffer maternal prenatal psychosocial stressors because such stressors relate to offspring fitness. Our previous work found that greater levels of emotional social support from and communication with maternal grandmothers, over and above father involvement, was related to lower prenatal psychological distress for mothers. Here, we extend this argument beyond assessing if mothers’ allomother relationships correlate with reduced psychological distress to investigate if these relationships directly buffer maternal stressors (Figure 1B).

A psychosocial stressor often studied in this pathway is racial/ethnic discrimination (henceforth ‘discrimination’), or how people are unjustly grouped and differentially treated by society. While race/ethnicity is not biological, issues related to discrimination have long been recognised for their association with increased psychological distress and physical health (Pascoe & Richman, 2009; Williams et al., 2003). Depression was found to mediate the effect of discrimination on physical health among Latino immigrants (Cariello et al., 2022). Recent work further suggests a causal link; for example, increases in discrimination over time are linked to higher rates of psychological distress and depression, but not the other way around (Williams et al., 2018).

Here, we analyse data from a cohort of pregnant Latina women in Southern California to ask if allomothers buffer the prenatal psychological distress associated with discrimination. Latinos are the largest demographic group in California (Public Policy Institute of California, 2020), and often experience unique institutional barriers and psychosocial stressors. Institutional barriers (such as limited access to economic and social resources like higher education and equitable healthcare) and psychosocial stressors (such as discrimination and mistreatment in healthcare) are often due to institutional racism and cultural incompetency of people in power (American Psychiatric Association, 2017; Santos et al., 2021). For foreign-born Latinos, language barriers and concerns about deportation are also common psychosocial stressors (American Psychiatric Association, 2017).

Unfortunately, a salient experience for many Latinos living in the US is ethnic discrimination. Hence, we chose to focus on discrimination because of its strong implication in the DOHaD literature and the Latino-American experience. In a PEW research study conducted in 2021, 54% of Latinos reported experiencing some discrimination event in the last 12 months, with the number higher for certain sub-groups (PEW Research Center, 2021). Frequent perceptions of discrimination, including everyday discrimination, has been shown to be a significant source of stress (Williams & Mohammed, 2009). Recent events in US politics have also led to increased discrimination and negative sentiment against Latinos as well as increased psychological distress among Latinos living in the US (Fox, 2022; PEW Research Center, 2018). Examples of recent political events and circumstances that could detrimentally influence Latino mental health include negative statements against Mexican Americans and other Latino groups by US President Donald Trump during his 2016 campaign and term as president, the growth of White-nationalist terrorist groups, as well as increasingly aggressive rhetoric regarding immigration at the border leading to the inhumane treatment of Latino migrants at the US–Mexico border. Our research question is particularly meaningful for our cohort of Latina women owing to (1) the unique forms of discrimination in the US and (2) the high rates of perinatal mood disorders and adverse birth outcomes among Latinas.

Latina women experience rates of psychological distress 20% higher than non-Latina White women (CDC, 2019) and a disproportionate burden of depression during and after pregnancy. Latina women living in the US report elevated rates of postpartum depression compared with non-Latina White women (46.8% compared with 31.3%; Howell et al., 2005). These disparities are similarly pronounced for depression during pregnancy (Gavin et al., 2011), although prenatal depression is studied less often than postnatal. Rates of maternal depression are also impacted by generational status; second-generation (US-born) Latina women experience substantially higher depression (44%) compared with first-generation (foreign-born) Latina women (34%; Huang et al., 2007). More exposure to ethnic discrimination during pregnancy is associated with more depressive symptoms longitudinally across pregnancy among an ethnically diverse cohort (Noroña-Zhou et al., 2022), suggesting that pregnancy is a vulnerable period throughout gestation and that discrimination effects are cumulative.

Psychological distress can become embodied or ‘get under the skin’, which contributes to health disparities between minority and majority ethnic groups (Gravlee, 2009). For pregnant women, embodiment of adverse experiences can influence stress physiology and, in turn, fetal development, birth timing and infant outcomes (Path A in our conceptual model, Figure 1A). Indeed, connections between discrimination and greater cortisol during pregnancy, lower offspring birth weight, shorter gestation length and greater stress reactivity in infants have been repeatedly observed (Carty et al., 2011; Collins et al., 2004; Thayer et al., 2019) (Path A2). Previous studies cast discrimination as a robust predictor of Latina perinatal psychological health and the trajectories of health for the next generation (Paths A1 and A3). Specifically for Latinas, greater levels of discrimination were associated with elevated rates of depression and anxiety among 150 pregnant Latinas, at 24–32 weeks’ gestation and 4–6 weeks postpartum, while controlling for acculturation, acculturative stress and economic hardship (Santos et al., 2021). In a multisite cohort study of approximately 2000 Latina and Black women, those who experienced higher levels of discrimination were more likely to give birth prematurely compared with those who experienced discrimination less than once per year (Fryer et al., 2020). Discrimination during pregnancy has been associated with increased odds of low birth weight, mediated by depression among a two-thirds Latina cohort (Earnshaw et al., 2013). Analysis of multi-generational birth records reveals that any advantages of foreign-born birth weight, compared with the non-Latino White majority, disappear in subsequent generations and disappear more rapidly among Black Americans than Latino Americans (Andrasfay & Goldman, 2020; Collins et al., 2002), further suggesting that it is the exposure to socio-economic disadvantage and discrimination that creates a birthweight disparity between ethnic groups. Thus, this particular cohort (Latina pregnant women) and psychosocial stressor (ethnic discrimination) are a well-documented backdrop against which to ask our allomothering research question.

Humans have a flexible roster of kin and non-kin who help the mother–child dyad (Kramer, 2010). Grandmothers are often critical allomothers perinatally because of their experience, knowledge and availability owing to their own non-reproductive phase of life. Most studies on grandmother inclusive fitness suggest that the benefits to grandchild survivorship are mostly apparent at weaning age (Hawkes et al., 1998; Meehan et al., 2013). Other scholars have extended the window of critical grandmaternal care to the perinatal period including during breastfeeding, at birth and during pregnancy (Knorr & Fox, 2023; Myers et al., 2021; Rosenberg & Trevathan, 2002; Scelza & Hinde, 2019). While variation exists and is ecologically dependent (Hill & Hurtado, 2009; Strassmann & Kurapati, 2010), maternal grandmothers (MGMs) are associated with grandchild health benefits more consistently than paternal grandmothers (PGMs) (Chapman et al., 2021; Coall & Hertwig, 2010; Nenko et al., 2021; Sear & Mace, 2008; Strassmann & Garrard, 2011). While the maternal grandfather may also play a support role during pregnancy, we chose to focus on the grandmothers owing to the shared reproductive experiences of women.

Motivated by the lack of research on allocare during pregnancy, we previously found that maternal, but not paternal, grandmother social support (specifically, emotional support) and communication in the same cohort had a direct and positive effect on pregnant women's mental health (Knorr and Fox, 2023). That was the first paper, to our knowledge, that took an overtly evolutionary lens on social support during pregnancy. This paper, in contrast, considers the stress-buffering potential of different allomothers during pregnancy.

Our research question is salient to our cohort of Latina women living in the US as many cultures within the ‘Latino’ ethnic category share a prioritisation to family in decision-making and deference domains – a concept known as *familismo* (Sabogal et al., 1987). A longitudinal study among Mexican-American women living in Arizona found that values of *familismo* appeared to buffer the harmful effects of discrimination on depression, while neighbourhood cohesion did not (Curci et al., 2022). We therefore can focus on the role of family specifically over other social units (like neighbourhood). Emotional support from family has been shown to buffer psychological distress associated with high levels of everyday discrimination experienced by Asian Americans (Mossakowski & Zhang, 2014). Among Mexican-American adults living in California, the negative physical health effects of discrimination were only seen among the group where instrumental social support was low; additionally, the number of family members in the US was also protective against the ill-effects of discrimination (Finch & Vega, 2003). Based upon previous postnatal allomaternal research and our previous findings of a prenatal allomaternal effect, we predict that MGMs will exert a positive effect on maternal mental health, and the relationship of maternal discrimination with negative mental health will be attenuated for those mothers who have higher levels of MGM emotional support, communication, and geographic proximity (Pathway B1 in Figure 1). Similarly based on previous findings, we predict weaker effects for PGMs’ influence buffering maternal prenatal psychological distress.

We analyse the grandmother relationship characteristics of emotional support, communication and geographic proximity, respective to the pregnant mother, because they capture unique parts of the grandmother–mother relationship. Emotional support is a critical component of psychological stress-buffering (Turner & Marino, 1994). Generally, the construct of social support measures the care provided by or potentially available from a known individual, which can come in many forms including emotional, informational and instrumental (Dunkel Schetter & Brooks, 2009). We chose an emotional support metric for several reasons. Firstly, previous studies have shown that instrumental and emotional support have different effects on perinatal outcomes (Bedaso et al., 2021; Emmott & Mace, 2015). Secondly, emotional support has been extensively tied to stress-buffering in pregnant cohorts (Bedaso et al., 2021; Seguin et al., 1995), while instrumental support has been shown to buffer stress and improve emotional well-being during pregnancy only when emotional support was also present (Morelli et al., 2015). Additionally, our measure of social support is a subjective measure that asks how satisfied the pregnant mother is with the emotional support she receives. A subjective measure of support allows us to describe how much help a woman receives above or below expected amounts of emotional support. Therefore, the difference in how much maternal and paternal grandmothers may be expected to help is already included in the models as it is measured by the participant's own assessment.

During pregnancy, women exhibit unique psychological responses and sensitivities to stressors, justifying the need to examine this effect in the context of pregnancy (Slade et al., 2009). Additionally, the relationships between discrimination, emotional support and mental health during pregnancy must be examined carefully because of the complex interactions between these constructs. For example, African-American pregnant women who reported greater experiences of discrimination (owing to race, gender, age and education) also reported lower levels of social support (Dailey, 2009; specifically, Dailey uses the ‘Interpersonal Relationship Inventory’ which assesses emotional support). In contrast, Giurgescu et al. (2017) find that while social support and discrimination were independently associated with psychological well-being in a cohort of 107 African-American pregnant women, there was no significant interaction of these two variables on psychological well-being. Social support in Giurgescu et al. is assessed by the Medical Outcomes Study Social Support Survey, which is built to be a general metric of many domains of social support but includes many emotional support items. Thus, more research is needed for understanding the influence of discrimination and various domains of social support on psychology in pregnancy among different groups of people.

Geographic proximity is a variable used in much of the classic grandmother literature to proxy grandmother involvement (Callaghan et al., 2006; Chapman et al., 2018; Engelhardt et al., 2019; Eshete et al., 2019; Kemkes-Grottenthaler, 2005; Madrigal & Meléndez-Obando, 2008; Voland & Beise, 2002). Greater geographic proximity between grandmother–mother–offspring is usually associated with better mother–offspring outcomes (Chapman et al., 2018; Engelhardt et al., 2019; Sear & Mace, 2008; Strassmann & Garrard, 2011). In historical and non-market integrated societies (i.e. societies that practice foraging, pastoralism, or other forms of subsistence living), greater proximity is often a strong predictor of social support generally. However, in market-integrated societies, many types of social support do not depend on geographic location; for example, emotional, informational and financial support can be exchanged at great distances through phone or internet connections. Therefore, our study implementing a comparison of geographic proximity and communication allows us to see a clearer picture of grandmother–mother relationships since geographic proximity reflects physical availability and instrumental support and communication reflects emotional and informational support.

Greater levels of family communication among adult, immigrant Mexican women were associated with greater levels of perceived emotional support (Vega et al., 1991). However, communication itself is not inherently positive and can reflect and exacerbate negative relationships. Verbal conflict among grandmother–mother relationships has been associated with negative parenting and child behaviour outcomes (Barnett et al., 2012). We measure communication levels broadly, so both positive and negative allomother interactions are possible. We capture how communication may influence maternal psychology in either direction.

Latinos share high rates of both multigenerational residence (grandmothers and grandchildren living in the same home) (PEW Research Center, 2010) as well as, conversely, high rates of geographic separation owing to their being the largest immigrant group in the US (PEW Research Center, 2020). This built-in contrast of close and far proximity is a further boon to our study. Overall, our research question integrates DOHaD frameworks and evolutionary theory. At the same time, our question addresses how and through what means family networks support pregnant women during vulnerable periods and in vulnerable situations.

## 2. Methods

### 2.1. Cohort

This study emerges from Wave 1 of Mothers’ Cultural Experiences (MCE) study, a multi-site cohort among whom data were collected in 2016–2018 to answer research questions surrounding culture, stress and mental health. This project used an observational study design with self-report survey data from 361 pregnant and postpartum women. Women were approached in clinic waiting rooms across four sites in Southern California and asked if they met the following eligibility criteria: (1) self-identified as Latina, Chicana, Hispanic, or Mexican; (2) spoke English or Spanish; (3) were pregnant or recently postpartum; and (4) 18 years of age or older. Participants answered a 30–45-minute questionnaire independently and were compensated $10. Data is not publicly available because participants did not consent to sharing individual-level data publicly.

For this study, women who did not meet the eligibility criteria (*N* = 13) did not receive social support scales in their survey versions (*N* = 87), and those who were postnatal (*N* = 104) were removed before analysis leaving a final analytic cohort of *N* = 216 (some overlap of disqualifications). The 87 women who did not receive social support scales were the first women recruited in the MCE study, receiving Version 1.0 of the survey. Several measures were added with the implementation of Version 2.0 in order to maximise the number of constructs assessed. We dropped these earliest participants from analyses here in order to avoid non-random missingness. Since our research question involves expanding allocare research into the realm of pregnancy, we did not analyse data of postnatal participants.

### 2.2. Variable operationalisation

#### Predictor variable: discrimination

We used the validated Everyday Discrimination Scale as the primary predictor in both models, with instructions to consider each question in relation to their ethnicity/race (Williams et al., 1997). This scale has been cross-analysed in different ethnic groups and genders (Lewis et al., 2012) finding overall similarities except differential item functioning for one to two items depending on ethnic group. Since all participants self-identified as part of the same ethnic group, differential item functioning is unlikely to be an issue here. Indeed, we find overall high reliability of the discrimination measure with an overall Cronbach alpha (*α*) of 0.90. This Cronbach *α* is 0.91 for English-speaking participants (*α*E) and 0.86 for Spanish-speaking participants (*α*S). For a break-down of the items and values in all validated scales used in this study, see the Supplementary Material.

#### Outcome variables: maternal prenatal mental health

The maternal mental health outcome variables are operationalised with the following validated questionnaire-based instruments: Edinburgh Perinatal Depression Scale (Cox et al., 1987), Spielberger State-Trait Anxiety Inventory – Short Form (Marteau & Bekker, 1992) and the Shortened Perceived Stress Scale (Cohen et al., 1983). We find the following reliabilities for depression *α*= 0.85 (*α*E = 0.86, *α*S = 0.84); state-anxiety *α* = 0.81 (*α*E = 0.84, *α*S = 0.77); and perceived stress *α* = 0.52 (*α*E = 0.86, *α*S = 0.33). Owing to the low PSS Cronbach's *α* in the Spanish version, post-hoc, we repeated the PSS models separately for the cohort subsets who took the survey in English and Spanish and did not find evidence of differential effects by language (see the Supplementary Material).

#### Predictor variables: allomother relationships characteristics

We define family roles based on each individual's relationship to the fetus. Although the relationships are not typically titled this way until after the offspring is born, for clarity we will describe relationships from the perspective of the fetus: MGM, father and PGM. Maternal grandmother in this study is the mother who raised the pregnant participant. While a biological relationship was not required for our analysis, only 3.3% of MGMs were reported as not the biological mother to our participants (Table 1). Similarly, we define the fetus's father as who the participant felt would be the main father figure; in all cases, this was either her current relationship partner or the biological father. In our cohort, 87% of participants were in a relationship, 95% of which were with the baby's father (Table 1). The PGM is who participants identified as their baby's father figure's mother. Instructions to the participants clarified that this might be the participant's mother-in-law, boyfriend's mother or someone else.

**Table 1.** Demographics of the study cohort and descriptive statistics of the measures used in this study. SD: standard deviation. See SM for explanation of clinically significant cut-off scores for depression.

	Total (N=216)
**Age**	
Mean (SD)	29.8 (6.12)
Median [Min, Max]	29.4 [18.1, 45.3]
Missing	13 (6.0%)
**In A Relationship?**	
Yes	188 (87.0%)
No	23 (10.6%)
Missing	5 (2.3%)
**Parity**	
Nulliparous	71 (32.9%)
Parous	138 (63.9%)
Missing	7 (3.2%)
**Trimester**	
First	17 (7.9%)
Second	47 (21.8%)
Third	131 (60.6%)
Missing	21 (9.7%)
**Education**	
Less than High School	30 (13.9%)
High School or Equivalent	142 (65.7%)
More than High School	36 (16.7%)
Missing	8 (3.7%)
**Food Insecure**	
Food secure	110 (50.9%)
Food insecure	82 (38.0%)
Missing	24 (11.1%)
**Everyday Ethnic Discrimination**	
Mean (SD)	0.771 (0.647)
Missing	14 (6.5%)
**Depression (EPDS) (full scale range)**	
Mean (SD)	5.71 (4.71)
Missing	8 (3.7%)
**State Anxiety (full scale range)**	
Mean (SD)	1.68 (0.585)
Missing	11 (5.1%)
**Perceived Stress (full scale range)**	
Mean (SD)	5.16 (2.57)
Missing	9 (4.2%)
**Social Support from baby's Maternal Grandmother**	
Mean (SD)	2.65 (0.579)
Missing	15 (6.9%)
**Social Support from baby's Paternal Grandmother**	
Mean (SD)	2.10 (0.778)
Missing	29 (13.4%)
**Social Support from baby's father**	
Mean (SD)	2.71 (0.590)
Missing	9 (4.2%)
**Do you know who your baby's biological father is (or probably is)?**	
Yes	199 (92.1%)
No	9 (4.2%)
Missing	8 (3.7%)
**Is your baby's biological father your current relationship partner?**	
Yes	178 (82.4%)
No	5 (2.3%)
Does not apply, not in a romantic relationship	24 (11.1%)
Missing	9 (4.2%)

To measure each allomother's emotional support, we used the validated Multidimensional Scale of Perceived Social Support (Zimet et al., 1988), which measures an individuals’ general perception of acceptable levels of social support from different sources. For this study, we adapted the four-item family sub-scale of Multidimensional Scale of Perceived Social Support to refer specifically to the baby's grandmothers and father. Therefore, participants were asked to rate social support from specific individuals on a three-point Likert rating on four statements (e.g. ‘I get the emotional help and support I need from my mother’), which was then averaged (Zimet et al., 1988). Since half of the items directly reflect emotional support and the more general items did not change the mean scores when removed, we conclude that the scale is primarily a reflection of emotional support (see Supplementary Material in this paper and in (Knorr & Fox, 2023). Our data showed a high internal reliability for the emotional support scale adapted for the MGM *α* = 0.94 (*α*E = 0.94, *α*S = 0.93), PGM *α* = 0.94 (*α*E = 0.95, *α*S = 0.93) and father *α* = 0.97 (*α*E = 0.97, *α*S = 0.97).

Communication and geographic proximity were each operationalised based on one question: ‘How nearby does your [mother/baby's father/baby's father's mother] live?/¿Qué tan cerca vive su [madre/padre de su bebe/madre del padre de su bebé]?’ and ‘How often do you communicate with your [mother/baby's father/baby's father's mother]?/¿Qué tan seguido se comunica con su [madre/padre de su bebe/madre del padre de su bebé]?’ For each question, participants were offered a list of options. For communication, options were 1 = every day, 2 = more than once a week, 3 = more than once a month, 4 = once a month or less and 5 = never. We reverse-coded so that greater numbers indicated greater levels of communication. For geographic proximity, options were 1 = in my home, 2 = in my neighbourhood, 3 = outside my neighbourhood but close enough to visit during the day and 4 = too far to visit during the day. We reverse-coded this variable so greater numerical values were associated with greater levels of geographic proximity.

#### Control variables

We included six covariates in each model: father relationship characteristics (i.e. father emotional support, communication and geographic proximity), maternal age, trimester, parity, foreign-born status and a composite measure of socio-economic status (SES). We take into account father relationship characteristics in order to ensure that any significant PGM association was not just a proxy for father involvement. We do not use relationship status because of high collinearity with father relationship characteristics, which was a higher priority control variable. For SES, we made a composite variable, used elsewhere (Fox, 2022; Knorr & Fox, 2023), that unitises and sums the MacArthur Subjective Socioeconomic Status Scale (Adler et al., 2000), maternal education (operationalised as ‘less than high school’, ‘high school or equivalent’ (examples of high school equivalency include receiving a General Education Diploma, which the US and Canada uses to certify attainment of knowledge equivalent to high school education) or ‘more than high school’ (including bachelor degrees or higher, some college, or a vocational degree)) and food security (2-Item Screen to Identify Families at Risk for Food Insecurity by Hager et al. (2010)). All other covariates used a single question (e.g. foreign-born status = ‘What country were you born in?/¿En que país nació?’). We control for SES, foreign-born status, maternal age, trimester and parity because these were suspected as possible confounders owing to previous studies demonstrating associations with mental health (Bottino et al., 2012; Campos et al., 2008; Freeman et al., 2016). These covariates may all theoretically influence the relationship and availability of different alloparents as well (Supplemental Figure S2). Despite the high comorbidity of depression and anxiety (Hirschfeld, 2001), we did not control for other mental health variables in our particular model because this could open backdoor paths and create confounding owing to collider variables (Supplemental Figure S1).

### 2.3. Statistical models

The overarching research question is: do allomothers buffer the association of stressors, such as discrimination, with maternal prenatal psychological distress? We answer this question with two sets of multiple linear regression models: (1) testing the association of discrimination and maternal prenatal psychological distress; and (2) testing how allomother relationship characteristics moderate the relationship between discrimination and maternal prenatal psychological distress. We expect the first set of models (henceforth, Set 1) to be significant as this is a well-established relationship in the literature. After replicating this finding in our dataset, we explore the second set of models (henceforth, Set 2) by adding an interaction term. This interaction term serves as a stress-buffering measure with three characteristics of the allomother–mother relationship: (a) emotional support, (b) communication and (c) geographic proximity. Each model in Set 2 assesses stress-buffering on three separate measures of psychological distress: depression, state-anxiety and perceived stress. All models control for the same list of covariates described above. All statistical models were conducted using RStudio and R version 4.0.3 and packages including tidyverse, ggplot2, mice, lmtest and car (Fox & Weisberg, 2019; van Buuren & Groothuis-Oudshoorn, 2011; Wickham, 2016; Wickham et al., 2019; Zeileis & Hothorn, 2002). For a complete list of packages used, see the code provided.

This study was pre-registered at https://osf.io/sn7e4.

*Model Set 1: is discrimination is associated with psychological distress?*


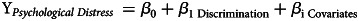




*Model Set 2: do allomother relationship characteristics moderate the association of discrimination and mental health?*


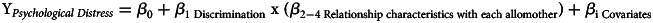




### 2.4. Missingness, imputation, power analysis and regression diagnostics

The analytic dataset had 7% overall missingness. In order to preserve sample size, we used multiple chain imputation with the MICE package in R, which uses group-level relationships between variables to impute five complete datasets (Section 2 in the Supplementary Material and Figure S3). The data are probably missing at random because the analytic cohort does not include any individuals who have systemic missingness (for post-hoc analysis surrounding our missing at random assumption, see Section 2.1 of the Supplementary Material).

We conducted regression analysis on each of the five imputed datasets and then pooled the output. Our models have 12 predictors (four variables of interest, five control variables and three interaction variables). With a sensitivity analysis for *F*-tests in G*Power (v3.1) and our parameters set to an *α*-error of 0.05 and a sample size of 216, our models can detect effect sizes down to 0.084 with 80% power (a medium effect size). Regression diagnostics, including Breusch–Pagan tests and variation inflation factor calculations, were run iteratively on all the models. These diagnostics are included in the Supplementary Material (Supplementary Tables S7–9). From these tests, we decided to use robust standard errors in all models to account for heteroscedasticity and remain conservative in our estimates.

## 3. Results

The women in our cohort were, on average, 29 years old (18–45 years old, standard deviation 6.12), in a relationship (87%), parous (63.9%), in their third trimester (60.6%) and educated with high school equivalency (66%) or less (14%) (Table 1). There was variation in the perceived levels of discrimination and psychological distress reported in this cohort, with high rates of self-reported depression (16.7%) and anxiety (22.7%) similar to what has been reported for Latina pregnant and postpartum women in other studies (Ponting et al., 2020). Additionally, Latinos disproportionately experience food insecurity in the US (16–22% compared with the national average of 11–14%; Rodriguez et al., 2021), although we find even higher levels of reported food insecurity among our cohort (38%).

### 3.1. Discrimination

**Figure 2.**
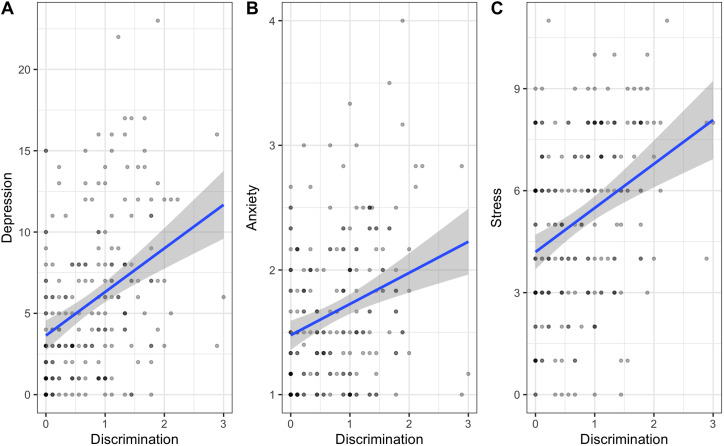
This figure shows the relationship between ethnic discrimination and depression (panel A), state anxiety (panel B), and perceived stress (panel C). The blue line represents the calculated beta slope from the regression model, while the gray shaded region represents the 95% confidence intervals of the estimates. These plots show non-pooled beta slopes from regressions using imputed dataset 2. We chose one dataset randomly, for clarity. The dots show up darker if there are multiple participants occupying that space.

In the first set of models, discrimination was a significant predictor of all three psychological distress measures. As predicted, higher self-reported levels of ethnic discrimination were associated with higher maternal depression (pooled *β*, 2.58; robust SE, 0.49; *p*-value < 0.001), state-anxiety (pooled *β*, 0.22; robust SE, 0.06; *p*-value < 0.001) and perceived stress (pooled *β*, 1.30; robust SE, 0.27; *p*-value < 0.001) (Table 2; Figure 2). Since these measures are all on unique scales the betas cannot be compared across models.

**Table 2.** Regression Results of Model Set 1 - The Relationship of Ethnic Discrimination and Prenatal Psychological Distress

	Depression	State Anxiety	Perceived Stress
Intercept	4.28	1.68***	3.77***
	(2.20)	(0.26)	(1.13)
Ethnic Discrimination	2.58***	0.22***	1.30***
	(0.49)	(0.06)	(0.27)
Socio-Economic Status	−0.97*	−0.09	−0.48*
	(0.43)	(0.05)	(0.23)
Age	0.01	0.00	0.01
	(0.06)	(0.01)	(0.03)
Trimester	0.26	−0.03	0.39
	(0.58)	(0.07)	(0.31)
Parity	−0.26	−0.02	−0.22
	(0.26)	(0.03)	(0.14)
Foreign Born	0.41	−0.12	0.26
	(0.63)	(0.08)	(0.37)
R-squared	0.17	0.10	0.15

The relationship of reported levels of everyday ethnic discrimination (row 2) on maternal depression, state-anxiety, and perceived stress (columns 1-3, respectively), holding certain covariates constant (row 3-7). Each cell contains the pooled beta, with stars indicating significance level (***p < 0.001; **p < 0.01; *p < 0.05) and pooled robust standard errors in the parentheses. Each model was run on a sample of N = 216. R^2^, the pooled coefficient of determination indicating how much variation in mental health is explained by the predictor and control variables, is also presented. Model comparison calculated from 5 imputed data sets against their respective null models produced the following pooled (F-statistics; p-values): depression (5.959; <0.0001), state anxiety (3.704; 0.001), perceived stress (5.314; <0.0001).

### 3.2. Grandmother stress-buffering

**Figure 3.**
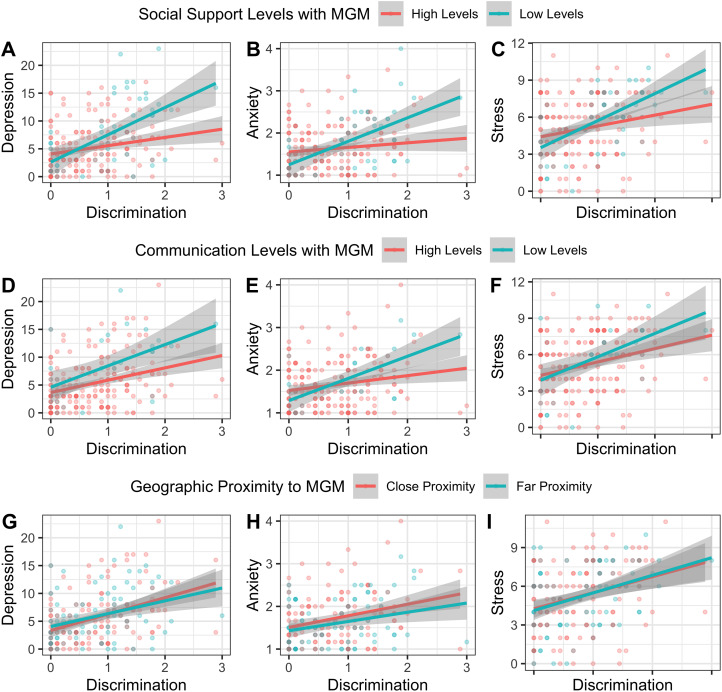
The How the interaction between allomother relationship characteristics and ethnic discrimination relates to prenatal psychological distress

In the second set of models, we evaluate how allomother relationship characteristics moderate the association between discrimination and psychological distress. As predicted, the interaction of MGM emotional support with discrimination moderated the relationship of discrimination and depression (pooled *β*, −1.61; robust SE, 0.73; *p*-value, 0.03) and of discrimination and anxiety (pooled *β*, −0.28; robust SE, 0.10; *p*-value, 0.005) (Table 3; Figure 3). Additionally, the interaction of MGM communication with discrimination was a significant moderator of discrimination and depression (pooled *β*, −0.78; robust SE, 0.39; *p*-value, 0.045), anxiety (pooled *β*, −0.14; SE, 0.05; *p*-value, 0.006), and stress (pooled *β*, −0.47; SE, 0.22; *p*-value, 0.035) (Table 4 and Figure 3). The PGM was not a significant moderator of any discrimination–psychological distress relationship. Geographic proximity of allomothers was not a significant buffer for any discrimination–psychological distress relationship (Table 5; Figure 3).

**Table 3.** Regression Results of Model Set 2 - How the interaction between emotional support and ethnic discrimination relates to prenatal psychological distress

	Depression	State Anxiety	Perceived Stress
Intercept	9.70*	1.65***	4.76*
	(3.73)	(0.45)	(2.01)
Ethnic Discrimination	7.02*	1.22***	3.56*
	(2.84)	(0.35)	(1.64)
Baby's Maternal Grandmother (MGM) - Emotional Support	0.30	0.20	0.47
	(0.91)	(0.11)	(0.52)
Baby's Paternal Grandmother (PGM) - Emotional Support	−0.79	−0.05	−0.42
	(0.69)	(0.09)	(0.41)
Baby's Father (F) - Emotional Support	−1.14	−0.08	−0.33
	(0.95)	(0.12)	(0.58)
Socio-Economic Status	−0.87*	−0.08	−0.44*
	(0.42)	(0.05)	(0.22)
Age	−0.02	0.00	−0.00
	(0.06)	(0.01)	(0.03)
Trimester	0.15	−0.05	0.34
	(0.53)	(0.06)	(0.30)
Parity	−0.30	−0.02	−0.23
	(0.25)	(0.03)	(0.13)
Foreign Born	0.26	−0.14	0.23
	(0.60)	(0.08)	(0.37)
Ethnic Discrimination × MGM Emotional Support	−1.61*	−0.28**	−0.69
	(0.73)	(0.10)	(0.43)
Ethnic Discrimination × PGM EmotionalSupport	0.60	0.00	0.57
	(0.64)	(0.09)	(0.36)
Ethnic Discrimination × F Emotional Support	−0.73	−0.12	−0.67
	(0.87)	(0.12)	(0.52)
R-squared	0.29	0.21	0.22

The moderation of emotional support from allomothers on ethnic discrimination (row 11-13) on depression, state-anxiety, and perceived stress (column 1-3, respectively), holding certain main effects (rows 2-5) and covariates (rows 6-10) constant. Each cell contains a pooled beta, with stars indicating the significance level (*** p < 0.001; ** p < 0.01; * p < 0.05) and robust standard errors in the parentheses. Each model was run on a sample of N = 216. R^2^ is also presented. Model comparisons calculated from 5 imputed data sets against their respective null models produced the following pooled F-statistics and p-values (respectively) for depression (5.760; <0.0001), state-anxiety (4.503; <0.0001), and perceived stress (3.563; <0.0001).

**Table 4.** Regression Results of Model Set 2 - How the interaction between communication and ethnic discrimination relates to prenatal psychological distress

	Depression	State Anxiety	Perceived Stress
Intercept	7.75*	1.28**	3.01
	(3.81)	(0.46)	(2.06)
Ethnic Discrimination	6.61*	1.01**	4.03*
	(2.75)	(0.35)	(1.60)
Baby's Maternal Grandmother (MGM) - Communication	0.01	0.12*	0.43
	(0.46)	(0.06)	(0.27)
Baby's Paternal Grandmother (PGM) - Communication	−0.38	−0.09	−0.29
	(0.45)	(0.05)	(0.25)
Baby's Father (F) - Communication	−0.28	0.05	0.01
	(0.51)	(0.07)	(0.30)
Socio-Economic Status	−1.02*	−0.10	−0.48*
	(0.42)	(0.05)	(0.23)
Age	−0.02	0.00	0.00
	(0.06)	(0.01)	(0.03)
Trimester	0.30	−0.03	0.37
	(0.55)	(0.07)	(0.30)
Parity	−0.32	−0.03	−0.23
	(0.26)	(0.03)	(0.14)
Foreign Born	0.29	−0.12	0.31
	(0.63)	(0.08)	(0.37)
Ethnic Discrimination × MGM Communication	−0.78*	−0.14**	−0.47*
	(0.39)	(0.05)	(0.22)
Ethnic Discrimination × PGM Communication	0.20	0.03	0.24
	(0.49)	(0.05)	(0.26)
Ethnic Discrimination × F Communication	−0.32	−0.07	−0.32
	(0.49)	(0.06)	(0.29)
R-squared	0.24	0.16	0.19

The moderation of communication from allomothers on ethnic discrimination (row 11-13) on depression, state-anxiety, and perceived stress (column 1-3, respectively), holding certain main effects (rows 2-5) and covariates (rows 6-10) constant. Each cell contains a pooled beta, with stars indicating the significance level (*** p < 0.001; ** p < 0.01; * p < 0.05) and robust standard errors in the parentheses. Each model was run on a sample of N = 216. R^2^ is presented. Model comparisons calculated from 5 imputed data sets against their respective null models produced the following pooled F-statistics and p-values (respectively) for depression (3.669; <0.0001), state-anxiety (2.655; 0.005), and perceived stress (2.788; 0.003).

**Table 5.** Regression Results of Model Set 2 - How the interaction between geographic proximity and discrimination relates to prenatal psychological distress

	Depression	State Anxiety	Perceived Stress
Intercept	3.35	1.43***	2.61
	(3.12)	(0.38)	(1.69)
Ethnic Discrimination	3.55	0.28	2.20
	(2.14)	(0.30)	(1.28)
Baby's Maternal Grandmother (MGM) - Geographic Proximity	0.12	0.07	0.19
	(0.40)	(0.05)	(0.22)
Baby's Paternal Grandmother (PGM) - Geographic Proximity	0.06	−0.08	0.14
	(0.54)	(0.07)	(0.28)
Baby's Father (F) - Geographic Proximity	0.08	0.03	0.01
	(0.51)	(0.07)	(0.28)
Socio-Economic Status	−0.95*	−0.10	−0.48*
	(0.43)	(0.05)	(0.23)
Age	0.02	0.01	0.02
	(0.07)	(0.01)	(0.03)
Trimester	0.20	−0.03	0.36
	(0.60)	(0.07)	(0.32)
Parity	−0.29	−0.03	−0.23
	(0.27)	(0.03)	(0.14)
Foreign Born	0.52	−0.08	0.44
	(0.68)	(0.09)	(0.39)
Ethnic Discrimination × MGM Geographic Proximity	−0.14	−0.03	−0.12
	(0.39)	(0.05)	(0.22)
Ethnic Discrimination × PGM Geographic Proximity	0.27	0.12	0.13
	(0.59)	(0.08)	(0.32)
Ethnic Discrimination × F Geographic Proximity	−0.32	−0.06	−0.25
	(0.48)	(0.06)	(0.25)
R-squared	0.18	0.14	0.17

The relationship of discrimination (row 2) on depression, state-anxiety, and perceived stress (columns 1-3, respectively), holding certain covariates constant (row 3-7). Each cell contains the pooled beta, with stars indicating significance level (*** p < 0.001; ** p < 0.01; * p < 0.05) and pooled robust standard errors in the parentheses. R^2^, the pooled coefficient of determination indicating how much variation in mental health is explained by the predictor and control variables, is also presented. Each model is run on a sample size of 216. Model comparison calculated from 5 imputed data sets against their respective null models produced the following pooled (F-statistics; p-values): depression (1.302, 0.230), state anxiety 1.039; 0.406), perceived stress (1.528; 0.133).

## 4. Discussion

Motivated by the epidemiological trends tying stressors to low-birth weight and preterm birth, we evaluate whether grandmothers buffer the psychological distress pregnant mothers experience from the stressor of racial/ethnic discrimination (henceforth discrimination) (Figure 1). In previous studies of postnatal allomothers, MGMs are shown to have a particular importance in offsetting the energetic expence of motherhood by providing childcare and direct provisioning of grandchildren during weaning (Hawkes et al., 1998; Meehan et al., 2013). Grandmaternal allocare also occurs during the prenatal period (Knorr & Fox, 2023). Here, we show that MGM can directly buffer the negative psychological response to stress during pregnancy. For Model Set 1, we replicate previous findings that discrimination is a significant stressor implicated in self-reported levels of depression, anxiety and perceived stress among pregnant women (Giurgescu et al., 2017; Mukherjee et al., 2016; Santos et al., 2021). For Model Set 2, we find that the relationship between maternal prenatal psychological distress and discrimination was moderated by greater levels of emotional support and communication with MGMs over and above any buffering effects of fathers. These results are consistent with the plausibility of the theoretical model presented in Figure 1, where MGMs are engaging in stress-buffering activities for pregnant mothers, potentially to improve the birth outcomes and later-in-life fitness outcomes of grandoffspring.

Research on grandmothers as critical allomothers often operationalises MGM and PGM help through proxies like grandmaternal survival, co-residence or geographic proximity, as these studies are most often conducted with historical records (e.g. Kemkes-Grottenthaler, 2005; Madrigal & Meléndez-Obando, 2008; Voland & Beise, 2002). Our work improves upon these studies by measuring a particular aspect of grandmother support more explicitly *in addition to* proxy variables like geographic proximity. This is similar to other postnatal studies that have also explored multiple domains of grandmothering (Emmott & Mace, 2015; Myers et al., 2021; Scelza & Hinde, 2019; Sheppard & Sear, 2016).

We predicted weaker effects for PGMs based on previous research. Consistent with this prediction, we find no evidence of PGM stress-buffering. This may be due to the absence of an effect or simply our analyses being underpowered to detect a smaller effect size compared with that of MGMs. The magnitude of each effect might be different for MGMs vs. PGMs given differences in expectations of help. Our emotional support measure is a subjective measure that asks women if they are satisfied with the level of support they are receiving from this person, so these differences in expectations of help are already included in the participant's assessment of social support. In contrast, geographic proximity and communication are objective measures, allowing us to harness both perspectives to compare different allomothers. Maternal grandmothers may be more consistent buffers of stress because mother–daughter relationships reflect a lifelong intimacy that is not comparable with mother-in-law relationships or even romantic relationships. Other cultural reasons may also account for this difference.

Since ethnic discrimination is such a rampant and distinct problem in large-scale, diverse societies like the US, these results may also be relevant to the minority health research discourse. Family relationships, specifically MGM–mother relationships, may play a particular role among Latina mothers, contributing to resilience against the ill effects of discrimination. This is an especially important public health application given that discrimination has been associated with adverse birth outcomes (Earnshaw et al., 2013; Fryer et al., 2020). Adverse birth outcomes, like low birthweight and preterm birth, are frequent causes of infant morbidity and mortality (Callaghan et al., 2006; Eshete et al., 2019). The developmental origins of health and disease framework explains how maternal psychological distress may modulate stress-related biochemical processes *in utero*, leading to adverse birth outcomes and affecting the offspring's lifespan disease-risk (including cardiovascular disease, obesity, diabetes, and psychopathology) (Thornburg & Marshall, 2015).

Family is a critical aspect to resilience. Individuals who report higher levels of *familismo* values tend to have greater levels of social support (as measured by the Medical Outcomes Study, a validated social support scale which reflects emotional support) as well as reduced risk of affective disorders (Campos et al., 2008). Our findings suggest that communication and emotional support are more critical buffers of discrimination than geographic proximity. This work suggests that encouraging strong social ties to community and extended family is important, supporting a broader anthropological discussion that an exclusively mother–father family unit is not better or even best (Sear, 2016). While in-person MGM care may be critical for instrumental support, these results show that maintaining positive relationships over the phone or internet could also have real and meaningful benefits.

## 5. Limitations

Our data does not include measures of fitness, such as number of children, grandchildren or offspring survivorship, so we make no claim of testing fitness. Additionally, our cross-sectional and observational design does not allow us to draw causal inferences. We also are limited by the scales used; for example, the discrimination scale does not ask about cumulative exposure to ethnic discrimination. Nor do we ask about intersectional experiences of discrimination, such as discrimination owing to gender or weight. Both cumulative exposures and the intersectional nature of identity can alter the experience of discrimination. We use the term ‘Latina’ to describe people who have been grouped together by wider socio-political power structures in addition to a specific set of cultural identities and experiences. Within this term, there exist many cultures and lifestyles, which we do not claim to fully capture.

While we suggest that emotional support is decoupled from geographic proximity and can act independently, we did not test whether certain types of remote communication were more effective for transmitting emotional support compared to others.

## 6. Conclusions

This study finds that discrimination is a significant stressor implicated in self-reported levels of depression, anxiety and perceived stress among pregnant Latina women. Additionally, we observe that MGMs buffer mothers’ psychological response to stress during pregnancy. This suggests that MGMs are likely participating in prenatal stress-buffering activities for mothers. Future work may connect these prenatal activities to improved birth outcomes and later-in-life fitness outcomes of grandoffspring through longitudinal study designs and the inclusion of infant outcome measures.

## Supporting information

Knorr and Fox supplementary material 1 Knorr and Fox supplementary material

Knorr and Fox supplementary material 2 Knorr and Fox supplementary material

Knorr and Fox supplementary material 3 Knorr and Fox supplementary material

## Data Availability

The data that support the findings of this study are not available because individuals only consented to sharing pooled results in publications, and not individual responses. The R code without the data is provided.

## Supplementary material

To view supplementary material for this article, please visit https://doi.org/10.1017/ehs.2023.27

## Research transparency and reproducibility

This paper was pre-registered at https://osf.io/sn7e4. For the statistical code, software, or other questions please contact the corresponding author.
