# Supplementary material for: Maternal grandmothers buffer the effects of ethnic discrimination among pregnant Latina mothers
Source: Evol Hum Sci. 2023 Nov 9;6:e7. doi: 10.1017/ehs.2023.27 (PMC10955360; doi:10.1017/ehs.2023.27)
Supplement: Knorr and Fox supplementary material 1 — Knorr and Fox supplementary material [file S2513843X23000270sup001.pdf]

R code for Publication

Code

Authors: Delaney A. Knorr [1], Molly Fox[1,2]

Affiliations:
[1] Department of Anthropology, University of California, Los Angeles, Los Angeles, CA 90095

[2] Department of Psychiatry & Biobehavioral Sciences, University of California Los Angeles, Los Angeles, CA 90095

Prep data, packages, and variables

Hide

```
# Set Working Directory  ---------------------------
setwd()

# Load libraries  ---------------------------
suppressMessages(library(tidyverse))
suppressMessages(library(mice))
suppressMessages(library(miceadds))
suppressMessages(library(table1))
suppressMessages(library(kableExtra))
suppressMessages(library(stargazer))
suppressMessages(library(sensemakr))
suppressMessages(library(knitr))
suppressMessages(library(mitml))
suppressMessages(library(broom.mixed))
suppressMessages(library(sandwich))
suppressMessages(library(lmtest))
suppressMessages(library(mitools))
suppressMessages(library(texreg))
suppressMessages(library(jtools))
suppressMessages(library(car))
suppressMessages(library(ggplot2))
suppressMessages(library(cowplot))
suppressMessages(library(gt))
suppressMessages(library(Amelia))
suppressMessages(library(ggpubr))
```

Hide

```
# Load data ---------------------------

imputed <- readRDS()  #to load data, add pathname within the readRDS function, dataset redacted for privacy of pa
rticipants

# Check imputation ---------------------------
mice::densityplot(imputed)
# the output of this graph is available in the Supplemental Materials (Supplemental Figure 3: Density Diagnostic
Plot of Imputed Variables)


# Load needed functions ---------------------------
na_count <- function (x) {
  na_count <- sapply(x, function(y) sum(length(which(is.na(y)))))
  na_count.df <- data.frame(na_count)
  myN <- sapply (x, function(y) sum(length(which(!is.na(y)))))
  df <- data.frame(na_count.df, myN)
  return(df)
}

normalize <- function(x) {
  x <- x - min(x, na.rm = T); x/max(x, na.rm = T);
}

extract.df <- function(tt, cl = NULL) {
  require(sandwich)
  require(mitools)
  require(texreg)
  m2 <- length(tt) #number of imputations
  betas <- lapply(tt, coef)
  vars <- lapply( tt, FUN=function(rr){ vcov(rr) } )
  # conduct statistical inference and save results into a data.frame
  model1 <- summary(pool_mi(betas, vars))

  R2 <- mean(sapply(1:m2, function(x) summary(tt[[x]])$r.squared))

  ns <- nobs(tt[[1]])
```

```
  #creates what is used by texreg
  tr <- createTexreg(
    coef.names = row.names(model1),
    coef = model1$results,
    se = model1$se,
    pvalues = model1$p,
    gof.names = c("R2", "Nobs"),
    gof = c(R2, ns),
    gof.decimal = c(T,F)
  )
}
```

Hide

```
# Generate new variables ---------------------------
# Change imputed datasets into format to manipulate
long <- complete(imputed, action='long', include=TRUE)
# Create SES composite variable
long$ses_full <- normalize(long$SES) + normalize(as.numeric(long$eduR)) + normalize(as.numeric(long$foodsecure))

#Create post-hoc binary variables for visualizations (Only significant variables were renamed at the end for visu
alizations)
#create and rename social support binary variable for all allomothers
#calculate median first
long$MomSocSuppBin <- ifelse(long$MomSocSupp >= 2.36, 1, 0)
long$MomSocSuppBin <- case_when(long$MomSocSuppBin == 1 ~ "High Levels",
                             long$MomSocSuppBin == 0 ~ "Low Levels")
long$MILSocSuppBin <- ifelse(long$MomSocSuppBin >= 4, 1, 0)
long$BFSocSuppBin <- ifelse(long$MomSocSuppBin >= 4, 1, 0)

#create and rename communication binary variable for all allomothers
long$MomCommBin <- ifelse(long$MomComm >= 4, 1, 0)
long$MomCommBin <- case_when(long$MomCommBin == 1 ~ "High Levels",
                             long$MomCommBin == 0 ~ "Low Levels")
long$MILCommBin <- ifelse(long$MILComm >= 4, 1, 0)
long$BFCommBin <- ifelse(long$BFComm >= 4, 1, 0)

#create and rename geographic proximity binary variable for all allomothers
median(df1$GeoproxR) # check what was the median of the non-imputed dataset
#above median split of 2 is Category 1
long$MomGeoBin <- ifelse(long$MomGeoProx >= 2, 1, 0)
long$MomGeoBin <- case_when(long$MomGeoBin == 1 ~ "Close Proximity",
                             long$MomGeoBin == 0 ~ "Far Proximity")
long$MILGeoBin <- ifelse(long$MILGeoProx >= 2, 1, 0)
long$BFGeoBin <- ifelse(long$BFGeoProx >= 2, 1, 0)
long$BFGeoBin <- case_when(long$BFGeoBin == 1 ~ "Close Proximity",
                             long$BFGeoBin == 0 ~ "Far Proximity")

#convert these maniputed variables back into the imputed dataset format
imputed <- as.mids(long)
```

```
# convert this imputed datalist into a format usable for table code (e.g., "Datlist")
datlist <- miceadds::mids2datlist(imputed)
```

Hide

```
# Table 1 Demographics ---------------------------

#load pre-imputed data
#[redacted]
#Missingness was either -99 (true missing) or -10 (prefer not to state)
# both forms of missingness are converted to NAs for consistency in reporting demographics
dfb4imp[dfb4imp == -99] <- NA
dfb4imp[dfb4imp == -10] <- NA

#check dimensions of dataset
dim(dfb4imp)

#Check dataset and convert predictor, outcome, and control variables to factors for Table 1
dat <- dfb4imp
glimpse(dat)
dat$Parity <- ifelse(dat$Parity == 0, 0, 1)

dat$Parity <- factor(dat$Parity,
                       levels=c(0,1),
                       labels=c("Nulliparous",
                                "Parous"))

dat$edu_ <- dplyr::recode(dat$eduR,
                            "1"="1",
                            "2"="2",
                            "3"="2",
                            "4"="3",
                            "5"="3",
                            "6"="3",
                            "7"="3",
                            "9"="3") #1 was <HS, 2 and 3 were HS , 4> more than HS

dat$Education <- factor(dat$edu_, levels=c(1,2,3),
                          labels=c("Less than High School",
                                   "High School or Equivalent",
                                   "More than High School"))

dat$RelStatus <-
```

```
  factor(dat$RelStatus, levels=c(1,0),
         labels=c("Yes",
                  "No"))

dat$Trimester <-
  factor(dat$tri, levels=c(1,2,3),
         labels=c("First",
                  "Second",
                  "Third"))

dat$FI <-
  factor(dat$foodsecure, levels=c(1, 0),
         labels=c("Food secure",
                  "Food insecure"))

label(dat$Parity) <- "Parity"
label(dat$RelStatus) <- "In A Relationship?"
label(dat$Age)     <- "Age"
units(dat$Age)       <- "years"

label(dat$ForBorn)     <- "Foreign Born"
label(dat$FI)     <- "Food Insecure"
label(dat$Education) <- "Education"


# Demographics of ppl born in other countries

dat$DHHCountry[which(dat$DHHCountry == "Another country")] <- dat$DHHCountryOther[which(dat$DHHCountry ==
 "Another country")]
dat$DHHCountry[which(dat$DHHCountry == "El salvador")] <- "El Salvador"
dat$DHHCountry[which(dat$DHHCountry == "Blank; doesn't apply")] <- NA
label(dat$DHHCountry) <- "Country of Origin"
#
table(dat$DHHCountry)


dat <- dat %>% mutate(country = case_when(DHHCountry == "U.S." ~ "U.S.",
                                    DHHCountry == "Mexico" ~ "Mexico",
                                    DHHCountry == "El Salvador" ~ "El Salvador",
```

```
                                     DHHCountry == "Guatemala" ~ "Guatemala",
                                     DHHCountry == "Brazil" ~ "Another country",
                                     DHHCountry == "Colombia" ~ "Another country",
                                     DHHCountry == "Honduras" ~ "Another country",
                                     DHHCountry == "Venezuela" ~ "Another country"
                                     ))
#
dat$country <- factor (dat$country, levels=c(
                "U.S.", "Mexico", "El Salvador", "Guatemala", "Another country"
              ))

label(dat$country) <- "Country of Origin"

# Mental Health Demographics
SA_fac <- ifelse(dat$CESTAISF > 2, 1, 0)
Depression_fac <- ifelse(dat$CEEPDS > 10, 1, 0)
# PSS_fac <- ifelse(dat$CEPSS > 8, 1, 0 ) #not a diagnostic scale so there are no cutoffs


dat$SA_fac <-
  factor(SA_fac, levels=c(1,0),
         labels=c("Anxious (>2)",
                  "Not Anxious"))

dat$Depression_fac <-
  factor(Depression_fac, levels=c(1,0),
         labels=c("Depressed (>10)",
                  "Not Depressed"))

label(dat$SA_fac) <- "State Anxiety (clinically significant symptoms)"
label(dat$Depression_fac) <- "Depression (clinically significant symptoms)"


label(dat$CEPSS) <- "Perceived Stress (full scale range)"
label(dat$CESTAISF) <- "State Anxiety (full scale range)"
label(dat$CEEPDS) <- "Depression (EPDS) (full scale range)"

# Communication
```

```
MomComm <- ifelse(dat$MomComm >= 4, 1, 0)
MILComm <- ifelse(dat$MILComm >= 4, 1, 0)
BFComm <- ifelse(dat$BFComm >= 4, 1, 0)


dat$MomComm <-
  factor(MomComm, levels=c(1,0),
         labels=c("Talks once a week or more",
                  "Talks less than once a week"))

dat$MILComm <-
  factor(MILComm, levels=c(1,0),
         labels=c("Talks once a week or more",
                  "Talks less than once a week"))


dat$BFComm <-
  factor(BFComm, levels=c(1,0),
         labels=c("Talks once a week or more",
                  "Talks less than once a week"))



label(dat$MomComm) <- "Communication Levels with baby's MGM"
label(dat$MILComm) <- "Communication Levels with baby's PGM"
label(dat$BFComm) <- "Communication Levels with baby's father"


# Geoproximity
MomGeo <- ifelse(dat$MomGeoProx >= 2, 1, 0)
MILGeo  <- ifelse(dat$MILGeoProx >= 2, 1, 0)
BFGeo  <- ifelse(dat$BFGeoProx >= 2, 1, 0)


dat$MomGeo <-
  factor(MomGeo, levels=c(1,0),
         labels=c("Lives in the same home or neighborhood",
                  "Lives in different neighborhood"))
```

```
dat$MILGeo <-
  factor(MILGeo, levels=c(1,0),
         labels=c("Lives in the same home or neighborhood",
                  "Lives in different neighborhood"))

dat$BFGeo <-
  factor(BFGeo, levels=c(1,0),
         labels=c("Lives in the same home or neighborhood",
                  "Lives in different neighborhood"))

label(dat$MomGeo) <- "Geographic Proximity to baby's MGM"
label(dat$MILGeo) <- "Geographic Proximity to baby's PGM"
label(dat$BFGeo) <- "Geographic Proximity to baby's father"

# Social Support

label(dat$MomSocSupp) <- "Social Support from baby's MGM"
label(dat$MILSocSupp) <- "Social Support from baby's PGM"
label(dat$BFSocSupp) <- "Social Support from baby's father"
```

Hide

```
# Add in other variables that weren' included in imputations calculations that are reported in demographics like genetic relationship to maternal grandmother and paternity certainty of father.

# Again, convert two types of missing answers for Table 1 clarity
df_full[df_full == -99] <- NA
df_full[df_full == -10] <- NA

#merge two datasets to get all the variables from just the SubIds we are interested in:
df_merged <- merge(df_full, dat, by = "SubID")
dim(df_merged)

# who is adopted?
table(df_merged$YSRMomRaise) # 2 'no one I think of as a mother'
which(df_merged$YSRMomRaise == "There is no one I think of as mother")
df_merged$SocSuppMom[c(X,Y)] #look at their data (relabeled as X and Y but these would be the # on their list)

which(df_merged$YSRMomRaise == "I was adopted and I'm not a blood relative of my parents")
df_merged$SocSuppMom[Z]
df_merged$MomCommR[Z]

dat$MomFig <- df_merged$YSRMomRaise
dat$MomLive <- df_merged$YSRMomLive
dat$BFLive <- df_merged$YSRDadLive
dat$MILLive <- df_merged$YSRFMomLive
dat$PatCert <- df_merged$YSRBbDad
dat$PatCert2 <- df_merged$YSRRelDad
dat$YSRDFathFig <- df_merged$YSRDFathFig

# Convert variables into factors for demographic tables
dat$MomFig <- factor (dat$MomFig, levels=c(
                "My birth mother raised me",
                "I was adopted and I'm not a blood relative of my parents",
                "Someone else I think of as a mother raised me"
              ))

dat$MomLive <- factor (dat$MomLive, levels=c(
                "U.S.",
                "Mexico",
                "Other"
```

```
              ))

dat$FathFig <- factor (dat$YSRDFathFig, levels=c(
                "My current partner",
                "The baby's biological father (not my current partner)",
                "Someone else"
              ))

dat$BFLive <- factor (dat$BFLive, levels=c(
                "U.S.",
                "Mexico",
                "Another country"
              ))

dat$MILLive <- factor (dat$MILLive, levels=c(
                "U.S.",
                "Mexico",
                "Another country"
              ))

dat$PatCert <- as.factor(ifelse(dat$PatCert == "No", 0,1))
dat$PatCert <- case_when (dat$PatCert  == "1" ~ "Yes",
                          dat$PatCert  == "0" ~ "No"
              )

dat$PatCert <- factor (dat$PatCert, levels=c(
                "Yes",
                "No"
              ))

dat$PatCert2 <- case_when (dat$PatCert2  == "Does not apply; they are not in a romantic relationship" ~ "Does not apply, not in a romantic relationship",
                           dat$PatCert2  == "Yes" ~ "Yes",
                           dat$PatCert2 == "No" ~"No"

              )
dat$PatCert2 <- factor (dat$PatCert2, levels=c(
                "Yes",
                "No",
```

```
                "Does not apply, not in a romantic relationship"
              ))

label(dat$age) <- "Age"
label(dat$MomFig) <- "Who do you think of as your mother?"
label(dat$MomLive) <- "Where does your mother (baby’s maternal grandmother) currently live?"
label(dat$MILLive) <- "Where does your baby's paternal grandmother currently live?"
label(dat$BFLive) <- "Where does your baby's father currently live?"
# label(dat$FathFig) <- "Who do you think of as your baby's father figure?"
label(dat$PatCert) <- "Do you know who your baby's biological father is (or probably is)?"
label(dat$PatCert2) <- "Is your baby's biological father, your current relationship partner?"
```

Hide

```
table1(~ age + RelStatus + Parity + Education + Trimester + FI +
         PatCert + PatCert2 +
         # FathFig +
         MomFig +
         CEEPDS + CESTAISF + CEPSS +
         Depression_fac + SA_fac +
         MomComm + MILComm + BFComm +
         MomGeo + MILGeo + BFGeo +
         MomLive + MILLive + BFLive +
         MomSocSupp + MILSocSupp + BFSocSupp, data=dat,
       caption = "Table 1: Demographics of the study cohort and descriptive statistics of the measures used in th
is study.
SD: standard deviation See SM for explanation of clinically significant cut-off scores.
", overall="Total", output = "latex")
```

Hide

```
# Mental Health Scales

#State Anxiety
stai.df.reverse <- df_merged %>% select(YFCalm, YFContent, YFRelaxed)

#write function to recode anxiety variables
recode.stai.reverse <- function(x){
  newvar <- dplyr::recode(x, "-99" = -99, "-10"=-10,"Not at all" =4,"Somewhat" = 3,"Moderately"=2,"Very much" =
1)
  return(newvar)
}

stai.df.reverse <- lapply(stai.df.reverse,recode.stai.reverse)
stai.df.reverse <- as.data.frame(lapply(stai.df.reverse,as.numeric))

stai.df <- df_merged %>% select(YFUpset, YFWorried, YFTense)

recode.stai <- function(x){
  newvar <- dplyr::recode(x, "-99" = -99, "-10"=-10,"Not at all" = 1,"Somewhat" = 2,"Moderately"=3,"Very much" =
4)
  return(newvar)
}

stai.df <- lapply(stai.df,recode.stai)
stai.df <- as.data.frame(lapply(stai.df,as.numeric))

stai.df <- cbind(stai.df,stai.df.reverse)

stai.df[stai.df==-99]<-NA
stai.df[stai.df==-10]<-NA

psych::alpha(stai.df)$total$std.alpha


# English
stai.df.reverse.eng <- df_merged %>% filter(Lang == "English") %>%
  select(YFCalm, YFContent, YFRelaxed)

#write function to recode anxiety variables
```

```
recode.stai.reverse.eng <- function(x){
  newvar <- dplyr::recode(x, "-99" = -99, "-10"=-10,"Not at all" = 4,"Somewhat" = 3,"Moderately"=2,"Very much" =
1)
  return(newvar)
}

stai.df.reverse.eng <- lapply(stai.df.reverse.eng,recode.stai.reverse.eng)
stai.df.reverse.eng <- as.data.frame(lapply(stai.df.reverse.eng,as.numeric))

stai.df.eng <- df_merged %>% filter(Lang == "English") %>% select(YFUpset, YFWorried, YFTense)

recode.stai.eng <- function(x){
  newvar <- dplyr::recode(x, "-99" = -99, "-10"=-10,"Not at all" = 1,"Somewhat" = 2,"Moderately"=3,"Very much" =
4)
  return(newvar)
}

stai.df.eng <- lapply(stai.df.eng,recode.stai.eng)
stai.df.eng <- as.data.frame(lapply(stai.df.eng,as.numeric))

stai.df.eng <- cbind(stai.df.eng,stai.df.reverse.eng)

stai.df.eng[stai.df.eng==-99]<-NA
stai.df.eng[stai.df.eng==-10]<-NA

psych::alpha(stai.df.eng)$total$std.alpha


#Spanish
stai.df.reverse.spa <- df_merged %>% filter(Lang == "Spanish") %>%
  select(YFCalm, YFContent, YFRelaxed)

#write function to recode anxiety variables
recode.stai.reverse.spa <- function(x){
  newvar <- dplyr::recode(x, "-99" = -99, "-10"=-10,"Not at all" = 4,"Somewhat" = 3,"Moderately"=2,"Very much" =
1)
  return(newvar)
}
```

```
stai.df.reverse.spa <- lapply(stai.df.reverse.spa,recode.stai.reverse.spa)
stai.df.reverse.spa <- as.data.frame(lapply(stai.df.reverse.spa,as.numeric))

stai.df.spa <- df_merged %>% filter(Lang == "Spanish") %>% select(YFUpset, YFWorried, YFTense)

recode.stai.spa <- function(x){
  newvar <- dplyr::recode(x, "-99" = -99, "-10"=-10,"Not at all" = 1,"Somewhat" = 2,"Moderately"=3,"Very much" =
4)
  return(newvar)
}

stai.df.spa <- lapply(stai.df.spa,recode.stai.spa)
stai.df.spa <- as.data.frame(lapply(stai.df.spa,as.numeric))

stai.df.spa <- cbind(stai.df.spa,stai.df.reverse.spa)

stai.df.spa[stai.df.spa==-99]<-NA
stai.df.spa[stai.df.spa==-10]<-NA

psych::alpha(stai.df.spa)$total$std.alpha


#Depression
depression.df <- df_merged %>% select(YFLaugh_Re, YFLookFor_Re, YFBlameMe_Re, YFAnxious_Re, YFPanic_Re, YFTopofme
_Re, YFDiffSleep_Re, YFSad_Re, YFCrying_Re, YFHarm_Re)

depression.df[depression.df==-99]<-NA
depression.df[depression.df==-10]<-NA

psych::alpha(depression.df)$total$std.alpha

# English
depression.df.eng <- df_merged %>% filter(Lang == "English") %>% select(YFLaugh_Re, YFLookFor_Re, YFBlameMe_Re, Y
FAnxious_Re, YFPanic_Re, YFTopofme_Re, YFDiffSleep_Re, YFSad_Re, YFCrying_Re, YFHarm_Re)

depression.df.eng[depression.df.eng==-99]<-NA
depression.df.eng[depression.df.eng==-10]<-NA

psych::alpha(depression.df.eng)$total$std.alpha
```

```
#Spanish
depression.df.spa <- df_merged %>% filter (Lang == "Spanish") %>% select(YFLaugh_Re, YFLookFor_Re, YFBlameMe_Re,
YFAnxious_Re, YFPanic_Re, YFTopofme_Re, YFDiffSleep_Re, YFSad_Re, YFCrying_Re, YFHarm_Re)

depression.df.spa[depression.df.spa==-99]<-NA
depression.df.spa[depression.df.spa==-10]<-NA

psych::alpha(depression.df.spa)$total$std.alpha

#PSS

pss.df <- df_merged %>% select(YFNoControl, YFDifficulties)
pss.df.r <- df_merged %>% select(YFConfident, YFYourWay)

recode.pss <- function(x){
  newvar <- dplyr::recode(x, "-99" = -99, "-10"=-10, "Never" = 0, "Almost never" = 1,"Sometimes" = 2,"Fairly ofte
n"=3,"Very often" = 4)
  return(newvar)
}

pss.df <- lapply(pss.df,recode.pss)
pss.df <- as.data.frame(lapply(pss.df,as.numeric))

recode.pss.r <- function(x){
  newvar <- dplyr::recode(x, "-99" = -99, "-10"=-10,"Never" = 4, "Almost never" = 3,"Sometimes" = 2,"Fairly ofte
n"=1,"Very often" = 0)
  return(newvar)
}

pss.df.r <- lapply(pss.df.r,recode.pss.r)
pss.df.r <- as.data.frame(lapply(pss.df.r,as.numeric))

pss.df <- cbind(pss.df.r,pss.df)


pss.df[pss.df==-99]<-NA
pss.df[pss.df==-10]<-NA
```

```
psych::alpha(pss.df)$total$std.alpha

#English

pss.df.eng <- df_merged %>% filter(Lang == "English") %>% select(YFNoControl, YFDifficulties)
pss.df.r.eng <- df_merged %>% filter(Lang == "English") %>% select(YFConfident, YFYourWay)

recode.pss.eng <- function(x){
  newvar <- dplyr::recode(x, "-99" = -99, "-10"=-10, "Never" = 0, "Almost never" = 1,"Sometimes" = 2,"Fairly ofte
n"=3,"Very often" = 4)
  return(newvar)
}

pss.df.eng <- lapply(pss.df.eng,recode.pss.eng)
pss.df.eng <- as.data.frame(lapply(pss.df.eng,as.numeric))

recode.pss.r.eng <- function(x){
  newvar <- dplyr::recode(x, "-99" = -99, "-10"=-10,"Never" = 4, "Almost never" = 3,"Sometimes" = 2,"Fairly ofte
n"=1,"Very often" = 0)
  return(newvar)
}

pss.df.r.eng <- lapply(pss.df.r.eng,recode.pss.r.eng)
pss.df.r.eng <- as.data.frame(lapply(pss.df.r.eng,as.numeric))

pss.df.eng <- cbind(pss.df.r.eng,pss.df.eng)



pss.df.eng[pss.df.eng==-99]<-NA
pss.df.eng[pss.df.eng==-10]<-NA

psych::alpha(pss.df.eng)$total$std.alpha

# Spanish

pss.df.spa <- df_merged %>% filter(Lang == "Spanish") %>% select(YFNoControl, YFDifficulties)
pss.df.r.spa <- df_merged %>% filter(Lang == "Spanish") %>% select(YFConfident, YFYourWay)

recode.pss.spa <- function(x){
```

```
  newvar <- dplyr::recode(x, "-99" = -99, "-10"=-10, "Never" = 0, "Almost never" = 1,"Sometimes" = 2,"Fairly ofte
n"=3,"Very often" = 4)
  return(newvar)
}

pss.df.spa <- lapply(pss.df.spa,recode.pss.spa)
pss.df.spa <- as.data.frame(lapply(pss.df.spa,as.numeric))

recode.pss.r.spa <- function(x){
  newvar <- dplyr::recode(x, "-99" = -99, "-10"=-10,"Never" = 4, "Almost never" = 3,"Sometimes" = 2,"Fairly ofte
n"=1,"Very often" = 0)
  return(newvar)
}

pss.df.r.spa <- lapply(pss.df.r.spa,recode.pss.r.spa)
pss.df.r.spa <- as.data.frame(lapply(pss.df.r.spa,as.numeric))

pss.df.spa <- cbind(pss.df.r.spa,pss.df.spa)


pss.df.spa[pss.df.spa==-99]<-NA
pss.df.spa[pss.df.spa==-10]<-NA

psych::alpha(pss.df.spa)$total$std.alpha


#social support multidimentional scales

recode.socsupp <- function(x){
  newvar <- dplyr::recode(x, "-99" = -99, "-10"=-10,"Not true" = 1, "Somewhat true" = 2,"Very true" = 3, "Does no
t apply" = -10) #
  return(newvar)
}

#socsupp mom
ssmom.df <- df_merged %>% select(YSRMomHelp, YSRMomEmo, YSRMomProb, YSRMomDec)

ssmom.df <- lapply(ssmom.df,recode.socsupp)
ssmom.df <- as.data.frame(lapply(ssmom.df,as.numeric))
```

```
ssmom.df[ssmom.df==-99]<-NA
ssmom.df[ssmom.df==-10]<-NA

psych::alpha(ssmom.df)$total$std.alpha

#english
ssmom.df.eng <- df_merged %>% filter(Lang == "English") %>% select(YSRMomHelp, YSRMomEmo, YSRMomProb, YSRMomDec)

ssmom.df.eng <- lapply(ssmom.df.eng,recode.socsupp)
ssmom.df.eng <- as.data.frame(lapply(ssmom.df.eng,as.numeric))

ssmom.df.eng[ssmom.df.eng==-99]<-NA
ssmom.df.eng[ssmom.df.eng==-10]<-NA

psych::alpha(ssmom.df.eng)$total$std.alpha

#spanish
ssmom.df.spa <- df_merged %>% filter(Lang == "Spanish") %>% select(YSRMomHelp, YSRMomEmo, YSRMomProb, YSRMomDec)

ssmom.df.spa <- lapply(ssmom.df.spa,recode.socsupp)
ssmom.df.spa <- as.data.frame(lapply(ssmom.df.spa,as.numeric))

ssmom.df.spa[ssmom.df.spa==-99]<-NA
ssmom.df.spa[ssmom.df.spa==-10]<-NA

psych::alpha(ssmom.df.spa)$total$std.alpha

#socsupp bf
ssbf.df <- df_merged %>% select(YSRDadHelp, YSRDadEmo, YSRDadProb, YSRDadDec)

ssbf.df <- lapply(ssbf.df,recode.socsupp)
ssbf.df <- as.data.frame(lapply(ssbf.df,as.numeric))

ssbf.df[ssbf.df==-99]<-NA
ssbf.df[ssbf.df==-10]<-NA

psych::alpha(ssbf.df)$total$std.alpha
```

```
#english
ssbf.df.eng <- df_merged %>% filter(Lang == "English") %>% select(YSRDadHelp, YSRDadEmo, YSRDadProb, YSRDadDec)

ssbf.df.eng <- lapply(ssbf.df.eng,recode.socsupp)
ssbf.df.eng <- as.data.frame(lapply(ssbf.df.eng,as.numeric))

ssbf.df.eng[ssbf.df.eng==-99]<-NA
ssbf.df.eng[ssbf.df.eng==-10]<-NA

psych::alpha(ssbf.df.eng)$total$std.alpha

#spanish
ssbf.df.spa <- df_merged %>% filter(Lang == "Spanish") %>% select(YSRDadHelp, YSRDadEmo, YSRDadProb, YSRDadDec)

ssbf.df.spa <- lapply(ssbf.df.spa,recode.socsupp)
ssbf.df.spa <- as.data.frame(lapply(ssbf.df.spa,as.numeric))

ssbf.df.spa[ssbf.df.spa==-99]<-NA
ssbf.df.spa[ssbf.df.spa==-10]<-NA

psych::alpha(ssbf.df.spa)$total$std.alpha

#socsupp mil
ssmil.df <- df_merged %>% select(YSRFMomHelp, YSRFMomEmo, YSRFMomProb, YSRFMomDec)


ssmil.df <- lapply(ssmil.df,recode.socsupp)
ssmil.df <- as.data.frame(lapply(ssmil.df,as.numeric))

ssmil.df[ssmil.df==-99]<-NA
ssmil.df[ssmil.df==-10]<-NA

psych::alpha(ssmil.df)$total$std.alpha #.94


#socsupp mil eng
ssmil.df.eng <- df_merged %>% filter(Lang == "English") %>%
  select(YSRFMomHelp, YSRFMomEmo, YSRFMomProb, YSRFMomDec)
```

```
ssmil.df.eng <- lapply(ssmil.df.eng,recode.socsupp)
ssmil.df.eng <- as.data.frame(lapply(ssmil.df.eng,as.numeric))

ssmil.df.eng[ssmil.df.eng==-99]<-NA
ssmil.df.eng[ssmil.df.eng==-10]<-NA

psych::alpha(ssmil.df.eng)$total$std.alpha #.95

#socsupp mil spanish
ssmil.df.spa <- df_merged %>% filter(Lang == "Spanish") %>%
  select(YSRFMomHelp, YSRFMomEmo, YSRFMomProb, YSRFMomDec)

ssmil.df.spa <- lapply(ssmil.df.spa,recode.socsupp)
ssmil.df.spa <- as.data.frame(lapply(ssmil.df.spa,as.numeric))

ssmil.df.spa[ssmil.df.spa==-99]<-NA
ssmil.df.spa[ssmil.df.spa==-10]<-NA

psych::alpha(ssmil.df.spa)$total$std.alpha #.94



# Discrimination
# no reverse scoring
recode.EDS <- function(x){
  newvar <- dplyr::recode(x, "-99" = -99, "-10"=-10,"Never" = 0, "Rarely" = 1,"Sometimes" = 2, "Often" = 3) #
  return(newvar)
}


eds.df <- df_merged %>% select(CELessRes, CEUnfair, CECritAccent, CENotSmart, CEAfraid, CEDishonest, CEBetterthan
you, CEThreat, CEFollStore)


eds.df.eng <- df_merged %>% filter(Lang == "English") %>% select(CELessRes, CEUnfair, CECritAccent, CENotSmart, C
EAfraid, CEDishonest, CEBetterthanyou, CEThreat, CEFollStore)


eds.df.spa <- df_merged %>% filter(Lang == "Spanish") %>% select(CELessRes, CEUnfair, CECritAccent, CENotSmart, C
```

```
EAfraid, CEDishonest, CEBetterthanyou, CEThreat, CEFollStore)


eds.df <- lapply(eds.df,recode.EDS)
eds.df <- as.data.frame(lapply(eds.df,as.numeric))

psych::alpha(eds.df)$total$std.alpha


eds.df.eng <- lapply(eds.df.eng,recode.EDS)
eds.df.eng <- as.data.frame(lapply(eds.df.eng,as.numeric))

psych::alpha(eds.df.eng)$total$std.alpha


eds.df.spa <- lapply(eds.df.spa,recode.EDS)
eds.df.spa <- as.data.frame(lapply(eds.df.spa,as.numeric))

psych::alpha(eds.df.spa)$total$std.alpha
```

Hide

```
# Supplemental Materials


Misstest <- dat %>% mutate(Depressed = ifelse(dat$Depression > 10, 1, 0))
na_count(Misstest)
rowSums(is.na(Misstest))
Misstest$na_count <- apply(Misstest, 1, function(x) sum(is.na(x)))

t.test(Misstest %>% filter(Depressed == 1) %>% select(na_count), Misstest %>% filter(Depressed == 0) %>% select(na_count))

t.test(Misstest %>% filter(ForBorn == 1) %>% select(na_count), Misstest %>% filter(ForBorn == 0) %>% select(na_count))
```

Results

Set 1: Discrimination on MH

Depression ~ Discrimination

Hide

```
depression_discrimination_nomoderation <- with(imputed, lm(CEEPDS ~ CEEDS +
                                                         I(normalize(SES) +
                                                           normalize(as.numeric(eduR)) +
normalize(as.numeric(foodsecure))) +
                                                         age + tri+ Parity + ForBorn))

summary(pool(depression_discrimination_nomoderation))
pool.r.squared(depression_discrimination_nomoderation) #pooled R^2 (not adjusted)


mod_depression_discrimination_nomoderation <- with(datlist, lm(CEEPDS ~ CEEDS +
                                                         ses_full +
                                                         age + tri+ Parity + ForBorn))

betas1 <- lapply( mod_depression_discrimination_nomoderation, FUN=function(rr){ coef(rr) } )
vars1 <- lapply( mod_depression_discrimination_nomoderation, FUN=function(rr){ vcov(rr) } ) #robust standard erro
rs
beta_names1 <- names(betas1[[1]])
cons1 <- beta_names1[-1]
summary(pool_mi(betas1, vars1))


mod1 <- extract.df(mod_depression_discrimination_nomoderation)
testEstimates(mod_depression_discrimination_nomoderation)
Ftest1 <- mitml::testConstraints( mod_depression_discrimination_nomoderation, constraints= cons1 )
print(Ftest1) # get F-test statistics of pooled models
```

Anxiety ~ Discrimination

Hide

```
# anxiety is not significant if adding other mental health variables....
anxiety_discrimination_nomoderation <- with(imputed, lm(CESTAISF  ~ CEEDS +
                                                         I(normalize(SES) +
                                                           normalize(as.numeric(eduR)) +
normalize(as.numeric(foodsecure))) +
                                                         age + tri+ Parity + ForBorn))

summary(pool(anxiety_discrimination_nomoderation))
pool.r.squared(anxiety_discrimination_nomoderation) #not adjusted R^2


#anxiety
mod_anxiety_discrimination_nomoderation <- with(datlist, lm(CESTAISF ~ CEEDS +
                                                         ses_full +
                                                         age + tri+ Parity + ForBorn))

betas2 <- lapply( mod_anxiety_discrimination_nomoderation, FUN=function(rr){ coef(rr) } )
vars2 <- lapply( mod_anxiety_discrimination_nomoderation, FUN=function(rr){ vcov(rr) } ) #robust standard errors
beta_names2 <- names(betas2[[1]])
cons2 <- beta_names2[-1]
summary(pool_mi(betas2, vars2))


mod2 <- extract.df(mod_anxiety_discrimination_nomoderation)
testEstimates(mod_anxiety_discrimination_nomoderation)
Ftest2 <- mitml::testConstraints( mod_anxiety_discrimination_nomoderation, constraints= cons2 )
print(Ftest2)
```

Stress ~ Discrimination

Hide

```
stress_discrimination_nomoderation <- with(imputed, lm( CEPSS~ CEEDS +
                                                         I(normalize(SES) +
                                                           normalize(as.numeric(eduR)) +
normalize(as.numeric(foodsecure))) +
                                                         age + tri+ Parity + ForBorn))

summary(pool(stress_discrimination_nomoderation))
pool.r.squared(stress_discrimination_nomoderation) #not adjusted R^2


mod_stress_discrimination_nomoderation <- with(datlist, lm(CEPSS ~ CEEDS +
                                                         ses_full +
                                                         age + tri+ Parity + ForBorn))

betas3 <- lapply( mod_stress_discrimination_nomoderation, FUN=function(rr){ coef(rr) } )
vars3 <- lapply( mod_stress_discrimination_nomoderation, FUN=function(rr){ vcov(rr) } ) #robust standard errors
summary(pool_mi(betas3, vars3))
beta_names3 <- names(betas3[[1]])
cons3 <- beta_names3[-1]


mod3 <- extract.df(mod_stress_discrimination_nomoderation)
testEstimates(mod_stress_discrimination_nomoderation)
Ftest3 <- mitml::testConstraints( mod_stress_discrimination_nomoderation, constraints= cons3 )
print(Ftest3)
```

Hide

```
output <- texreg::htmlreg(list(mod1,mod2,mod3), digits = 2, custom.model.names = c('Depression', 'State Anxiet
y','Perceived Stress'), custom.coef.names = c('Intercept','Discrimination','Socio-Economic Status', 'Age','Trimes
ter','Parity','Foreign Born'),table.margin = 0, center = TRUE, caption = "The relationship of discrimination (row
2) on depression, state-anxiety, and perceived stress (columns 1-3, respectively), holding certain covariates con
stant (rows 3-7). Each cell contains the pooled beta, with stars indicating significance level and pooled robust
standard errors in the parentheses. R2 and the total number of women in the study, are also presented.
Model comparison calculated from 5 imputed data sets against their respective null models produced the following
pooled (F-statistics; p-values): depression 5.959; <0.0001), state anxiety (3.704; <0.0001), pregnancy-related an
xiety (5.314; <0.0001). DOUBLE CHECK F-STATS BEFORE SUBMITTING
")

output
```

Hide

```
sample(1:5, 1) # choose random imputed dataset to run visualizations on
# chose 2, so dataset 2


mod <- lm(CEEPDS ~ CEEDS + ses_full + age + tri+ Parity + ForBorn, df2)
summary(mod)
mod <- lm(CESTAISF ~ CEEDS + ses_full + age + tri+ Parity + ForBorn, df2)
summary(mod)
mod <- lm(CEPSS ~ CEEDS + ses_full + age + tri+ Parity + ForBorn, df2)
summary(mod)

# Depression
d1 <- ggplot(df2,
       aes(x = CEEDS,
           y = CEEPDS)) +
  theme_bw() +
  labs(x = "Discrimination",
       y = "Depression") +
  geom_point(alpha = .3,
             size = .9) +
  geom_smooth(method = "lm")

#Anxiety

a1 <- ggplot(df2,
       aes(x = CEEDS,
           y = CESTAISF)) +
  theme_bw() +
  labs(x = "Discrimination",
       y = "Anxiety") +
  geom_point(alpha = .3,
             size = .9) +
  geom_smooth(method = "lm")

#Stress
s1 <- ggplot(df2,
       aes(x = CEEDS,
           y = CEPSS)) +
  theme_bw() +
```

```
  labs(x = "Discrimination",
       y = "Stress") +
  geom_point(alpha = .3,
             size = .9) +
  geom_smooth(method = "lm")

disc1 <- ggarrange(d1, a1, s1,
          labels = c("A", "B", "C"),
          ncol = 3, nrow = 1, align = "hv")

disc1
```

Set 2: Moderation of Discrimination on MH

Set 2a: Social Support Moderator

Depression

Hide

```
depression_discrimination_socsupp_moderation <- with(imputed, lm(CEEPDS ~ CEEDS + MomSocSupp + BFSocSupp + MILSoc
Supp +
                                                         I(normalize(SES) +
                                                           normalize(as.numeric(eduR)) +
normalize(as.numeric(foodsecure))) +
                                                         age + tri+ Parity + ForBorn))

summary(pool(depression_discrimination_socsupp_moderation))


depression_discrimination_socsupp_moderation <- with(imputed, lm(CEEPDS ~ CEEDS*(MomSocSupp + BFSocSupp + MILSocS
upp) +
                                                         I(normalize(SES) +
                                                           normalize(as.numeric(eduR)) +
normalize(as.numeric(foodsecure))) +
                                                         age + tri+ Parity + ForBorn))

summary(pool(depression_discrimination_socsupp_moderation))
pool.r.squared(depression_discrimination_socsupp_moderation)


#depression
mod_depression_discrimination_socsupp_moderation<- with(datlist, lm(CEEPDS ~ CEEDS*(MomSocSupp + BFSocSupp + MILS
ocSupp) +
                                                         ses_full +
                                                         age + tri+ Parity + ForBorn))



betas4 <- lapply( mod_depression_discrimination_socsupp_moderation, FUN=function(rr){ coef(rr) } )
vars4 <- lapply( mod_depression_discrimination_socsupp_moderation, FUN=function(rr){ vcov(rr) } ) #robust standar
d errors
summary(pool_mi(betas4, vars4))
beta_names4 <- names(betas4[[1]])
cons4 <- c(beta_names4)[2:10]


mod4 <- extract.df(mod_depression_discrimination_socsupp_moderation)
testEstimates(mod_depression_discrimination_socsupp_moderation)
```

```
Ftest4 <- mitml::testConstraints(mod_depression_discrimination_socsupp_moderation, constraints= cons4 )
print(Ftest4)
```

Anxiety

Hide

```
anxiety_discrimination_socsupp_moderation <- with(imputed, lm(CESTAISF ~ CEEDS*(MomSocSupp + BFSocSupp + MILSocSu
pp) +
                                                         I(normalize(SES) +
                                                           normalize(as.numeric(eduR)) +
normalize(as.numeric(foodsecure))) +
                                                         age + tri+ Parity + ForBorn))

summary(pool(anxiety_discrimination_socsupp_moderation))
pool.r.squared(anxiety_discrimination_socsupp_moderation)


mod_anxiety_discrimination_socsupp_moderation <- with(datlist, lm(CESTAISF ~ CEEDS*(MomSocSupp + BFSocSupp + MILS
ocSupp) +
                                                         ses_full +
                                                         age + tri+ Parity + ForBorn))



betas5 <- lapply( mod_anxiety_discrimination_socsupp_moderation, FUN=function(rr){ coef(rr) } )
vars5 <- lapply( mod_anxiety_discrimination_socsupp_moderation, FUN=function(rr){ vcov(rr) } ) #robust standard e
rrors
summary(pool_mi(betas5, vars5))
beta_names5 <- names(betas5[[1]])
cons5 <- beta_names5[2:10]


mod5 <- extract.df(mod_anxiety_discrimination_socsupp_moderation)
testEstimates(mod_anxiety_discrimination_socsupp_moderation)
Ftest5 <- mitml::testConstraints( mod_anxiety_discrimination_socsupp_moderation, constraints= cons5 )
#
print(Ftest5)
```

Stress

Hide

```
stress_discrimination_socsupp_moderation <- with(imputed, lm(CEPSS ~ CEEDS*(MomSocSupp + BFSocSupp + MILSocSupp)
+
                                                         I(normalize(SES) +
                                                           normalize(as.numeric(eduR)) +
normalize(as.numeric(foodsecure))) +
                                                         age + tri+ Parity + ForBorn))

summary(pool(stress_discrimination_socsupp_moderation))
pool.r.squared(stress_discrimination_socsupp_moderation)

mod_stress_discrimination_socsupp_moderation <- with(datlist, lm(CEPSS ~ CEEDS*(MomSocSupp + BFSocSupp + MILSocSu
pp) +
                                                         ses_full +
                                                         age + tri+ Parity + ForBorn))


betas6 <- lapply( mod_stress_discrimination_socsupp_moderation, FUN=function(rr){ coef(rr) } )
vars6 <- lapply( mod_stress_discrimination_socsupp_moderation, FUN=function(rr){ vcov(rr) } ) #robust standard er
rors
summary(pool_mi(betas6, vars6))
beta_names6 <- names(betas6[[1]])
cons6 <- beta_names6[2:10]


mod6 <- extract.df(mod_stress_discrimination_socsupp_moderation)
testEstimates(mod_stress_discrimination_socsupp_moderation)
Ftest6 <- mitml::testConstraints( mod_stress_discrimination_socsupp_moderation, constraints= cons6 )
print(Ftest6)
```

Hide

```
# Depression
d2_ss <- ggplot(df2,
       aes(x = CEEDS,
           y = CEEPDS,
           color = MomSocSuppBin)) +
  theme_bw() +
  labs(x = "Discrimination",
       y = "Depression",
       color = "Social Support Levels with MGM") +
  geom_point(alpha = .3,
             size = .9) +
  geom_smooth(method = "lm")

#Anxiety

a2_ss <- ggplot(df2,
       aes(x = CEEDS,
           y = CESTAISF,
           color = MomSocSuppBin)) +
  theme_bw() +
  labs(x = "Discrimination",
       y = "Anxiety",
       color = "Social Support Levels with MGM") +
  geom_point(alpha = .3,
             size = .9) +
  geom_smooth(method = "lm")

#Stress
s2_ss <- ggplot(df2,
       aes(x = CEEDS,
           y = CEPSS,
           color = MomSocSuppBin)) +
  theme_bw() +
  labs(x = "Discrimination",
       y = "Stress",
       color = "Social Support Levels with MGM") +
  geom_point(alpha = .3,
             size = .9) +
```

```
  geom_smooth(method = "lm")

SS <- ggarrange(d2_ss, a2_ss, s2_ss + rremove("x.text"), common.legend = TRUE,
          labels = c("A", "B", "C"),
          ncol = 3, nrow = 1)

SS
```

Hide

```
output2 <- texreg::htmlreg(list(mod4,mod5,mod6), digits = 2, custom.model.names = c('Depression', 'State Anxiet
y','Perceived Stress'), custom.coef.names = c('Intercept','Discrimination','MGM - Social Support','BF - Social Su
pport', 'PGM - Social Support', 'Socio-Economic Status', 'Age','Trimester','Parity','Foreign Born', 'Discrim*MGMS
S', 'Discrim*BFSS', 'Discrim*PGMSS'),table.margin = 0, center = TRUE, caption = "social support from allomothers
(rows 3-5) on relationship of discrimination (row 2) on depression, state-anxiety, and perceived stress (columns
1-3, respectively), holding certain covariates constant (rows 5-10). Each cell contains the pooled beta, with sta
rs indicating significance level and pooled robust standard errors in the parentheses. R2 and the total number of
women in the study, are also presented.
Model comparison calculated from 5 imputed data sets against their respective null models produced the following
pooled (F-statistics; p-values): depression XX; <0.0001), state anxiety (XX ; <0.0001), perceived stress (XX; <0.
0001).  DOUBLE CHECK F-STATS BEFORE SUBMITTING
")


output2
```

Set 2b: Geographic Proximity as Moderator

Depression

Hide

```
depression_discrimination_geoprox_moderation <- with(imputed, lm(CEEPDS ~ CEEDS*(MomGeoProx + BFGeoProx + MILGeoP
rox) +
                                                         I(normalize(SES) +
                                                           normalize(as.numeric(eduR)) +
normalize(as.numeric(foodsecure))) +
                                                         age + tri+ Parity + ForBorn))

summary(pool(depression_discrimination_geoprox_moderation))
pool.r.squared(depression_discrimination_geoprox_moderation)

#depression
mod_depression_discrimination_geoprox_moderation<- with(datlist, lm(CEEPDS ~ CEEDS*(MomGeoProx + BFGeoProx + MILG
eoProx) +
                                                         ses_full +
                                                         age + tri+ Parity + ForBorn))



betas7 <- lapply( mod_depression_discrimination_geoprox_moderation, FUN=function(rr){ coef(rr) } )
vars7 <- lapply( mod_depression_discrimination_geoprox_moderation, FUN=function(rr){ vcov(rr) } ) #robust standar
d errors
summary(pool_mi(betas7, vars7))
beta_names7 <- names(betas7[[1]])
cons7 <- c(beta_names7[2:10])



mod7 <- extract.df(mod_depression_discrimination_geoprox_moderation)
testEstimates(mod_depression_discrimination_geoprox_moderation)
Ftest7 <- mitml::testConstraints( mod_depression_discrimination_geoprox_moderation, constraints= cons7 )
print(Ftest7)
```

Anxiety

Hide

```
anxiety_discrimination_geoprox_moderation <- with(imputed, lm(CESTAISF ~ CEEDS*(MomGeoProx + BFGeoProx + MILGeoPr
ox) +
                                                         I(normalize(SES) +
                                                           normalize(as.numeric(eduR)) +
normalize(as.numeric(foodsecure))) +
                                                         age + tri+ Parity + ForBorn))

summary(pool(anxiety_discrimination_geoprox_moderation))
pool.r.squared(anxiety_discrimination_geoprox_moderation)


mod_anxiety_discrimination_geoprox_moderation <- with(datlist, lm(CESTAISF ~ CEEDS*(MomGeoProx + BFGeoProx + MILG
eoProx) +
                                                         ses_full +
                                                         age + tri+ Parity + ForBorn))


betas8 <- lapply( mod_anxiety_discrimination_geoprox_moderation, FUN=function(rr){ coef(rr) } )
vars8 <- lapply( mod_anxiety_discrimination_geoprox_moderation, FUN=function(rr){ vcov(rr) } ) #robust standard e
rrors
summary(pool_mi(betas8, vars8))
beta_names8 <- names(betas8[[1]])
cons8 <- beta_names8[2:10]


mod8 <- extract.df(mod_anxiety_discrimination_geoprox_moderation)
testEstimates(mod_anxiety_discrimination_geoprox_moderation)
Ftest8 <- mitml::testConstraints( mod_anxiety_discrimination_geoprox_moderation, constraints= cons8 )

print(Ftest8)
```

Stress

Hide

```
stress_discrimination_geoprox_moderation <- with(imputed, lm(CEPSS ~ CEEDS*(MomGeoProx + BFGeoProx + MILGeoProx)
+
                                                         I(normalize(SES) +
                                                           normalize(as.numeric(eduR)) +
normalize(as.numeric(foodsecure))) +
                                                         age + tri+ Parity + ForBorn))

summary(pool(stress_discrimination_geoprox_moderation))
pool.r.squared(stress_discrimination_geoprox_moderation)

mod_stress_discrimination_geoprox_moderation <- with(datlist, lm(CEPSS ~ CEEDS*(MomGeoProx + BFGeoProx + MILGeoPr
ox) +
                                                         ses_full +
                                                         age + tri+ Parity + ForBorn))


betas9 <- lapply( mod_stress_discrimination_geoprox_moderation, FUN=function(rr){ coef(rr) } )
vars9 <- lapply(mod_stress_discrimination_geoprox_moderation, FUN=function(rr){ vcov(rr) } ) #robust standard err
ors
summary(pool_mi(betas9, vars9))
beta_names9 <- names(betas9[[1]])
cons9 <- beta_names9[2:10]


mod9 <- extract.df(mod_stress_discrimination_geoprox_moderation)
testEstimates(mod_stress_discrimination_geoprox_moderation)
Ftest9 <- mitml::testConstraints( mod_stress_discrimination_geoprox_moderation, constraints= cons9 )
print(Ftest9)
```

Hide

```
output3 <- texreg::htmlreg(list(mod7,mod8,mod9), digits = 2, custom.model.names = c('Depression', 'State Anxiet
y','Perceived Stress'), custom.coef.names = c('Intercept','Discrimination','MGM - Geographic Prox.','BF - Geograp
hic Prox.', 'PGM - Geographic Prox.', 'Socio-Economic Status', 'Age','Trimester','Parity','Foreign Born', 'Discri
m*MGM GeoProx', 'Discrim*BF GeoProx', 'Discrim*PGM GeoProx'),table.margin = 0, center = TRUE, caption = "The mode
ration of geographic proximity from allomothers (rows 3-5) on relationship of discrimination (row 2) on depressio
n, state-anxiety, and perceived stress (columns 1-3, respectively), holding certain covariates constant (rows 5-1
0). Each cell contains the pooled beta, with stars indicating significance level and pooled robust standard error
s in the parentheses. R2 and the total number of women in the study, are also presented.")


output3
```

Set 2c: Communication as Moderator

Hide

```
depression_discrimination_comm_moderation <- with(imputed, lm(CEEPDS ~ CEEDS*(MomComm + BFComm+ MILComm) +
                                                         I(normalize(SES) +
                                                           normalize(as.numeric(eduR)) +
normalize(as.numeric(foodsecure))) +
                                                         age + tri+ Parity + ForBorn))

summary(pool(depression_discrimination_comm_moderation))
pool.r.squared(depression_discrimination_comm_moderation)


#depression
mod_depression_discrimination_comm_moderation<- with(datlist, lm(CEEPDS ~ CEEDS*(MomComm + BFComm+ MILComm) +
                                                         ses_full +
                                                         age + tri+ Parity + ForBorn))


betas10 <- lapply( mod_depression_discrimination_comm_moderation, FUN=function(rr){ coef(rr) } )
vars10 <- lapply( mod_depression_discrimination_comm_moderation, FUN=function(rr){ vcov(rr) } ) #robust standard
errors
summary(pool_mi(betas10, vars10))
beta_names10 <- names(betas10[[1]])
cons10 <- c(beta_names10[2:10])


mod10 <- extract.df(mod_depression_discrimination_comm_moderation)
testEstimates(mod_depression_discrimination_comm_moderation)
Ftest10 <- mitml::testConstraints(mod_depression_discrimination_comm_moderation, constraints= cons10)
print(Ftest10)
```

Hide

```
anxiety_discrimination_comm_moderation <- with(imputed, lm(CESTAISF ~ CEEDS*(MomComm + BFComm+ MILComm) +
                                                          I(normalize(SES) +
                                                            normalize(as.numeric(eduR)) +
normalize(as.numeric(foodsecure))) +
                                                          age + tri+ Parity + ForBorn))

summary(pool(anxiety_discrimination_comm_moderation))
pool.r.squared(anxiety_discrimination_comm_moderation)



mod_anxiety_discrimination_comm_moderation<- with(datlist, lm(CESTAISF ~ CEEDS*(MomComm + BFComm+ MILComm) +
                                                          ses_full +
                                                          age + tri+ Parity + ForBorn))


betas11 <- lapply( mod_anxiety_discrimination_comm_moderation, FUN=function(rr){ coef(rr) } )
vars11 <- lapply( mod_anxiety_discrimination_comm_moderation, FUN=function(rr){ vcov(rr) } ) #robust standard err
ors
summary(pool_mi(betas11, vars11))
beta_names11 <- names(betas11[[1]])
cons11 <- c(beta_names11[2:10])


mod11 <- extract.df(mod_anxiety_discrimination_comm_moderation)
testEstimates(mod_anxiety_discrimination_comm_moderation)
Ftest11 <- mitml::testConstraints( mod_anxiety_discrimination_comm_moderation, constraints= cons11 )
print(Ftest11)
```

Hide

```
stress_discrimination_comm_moderation <- with(imputed, lm(CEPSS ~ CEEDS*(MomComm + BFComm+ MILComm) +
                                                          I(normalize(SES) +
                                                            normalize(as.numeric(eduR)) +
normalize(as.numeric(foodsecure))) +
                                                          age + tri+ Parity + ForBorn))

summary(pool(stress_discrimination_comm_moderation))
pool.r.squared(stress_discrimination_comm_moderation)



mod_stress_discrimination_comm_moderation<- with(datlist, lm(CEPSS ~ CEEDS*(MomComm + BFComm+ MILComm) +
                                                          ses_full +
                                                          age + tri+ Parity + ForBorn))


betas12 <- lapply( mod_stress_discrimination_comm_moderation, FUN=function(rr){ coef(rr) } )
vars12 <- lapply( mod_stress_discrimination_comm_moderation, FUN=function(rr){ vcov(rr) } ) #robust standard erro
rs
summary(pool_mi(betas12, vars12))
beta_names12 <- names(betas12[[1]])
cons12 <- c(beta_names12[2:10])


mod12 <- extract.df(mod_stress_discrimination_comm_moderation)
testEstimates(mod_stress_discrimination_comm_moderation)
Ftest12 <- mitml::testConstraints( mod_stress_discrimination_comm_moderation, constraints= cons12 )
print(Ftest12)
```

Hide

```
output4 <- texreg::htmlreg(list(mod10,mod11,mod12), digits = 2, custom.model.names = c('Depression', 'State Anxie
ty','Perceived Stress'), custom.coef.names = c('Intercept','Discrimination','MGM - Communication','BF - Communica
tion ', 'PGM - Communication', 'Socio-Economic Status', 'Age','Trimester','Parity','Foreign Born', 'Discrim*MGM C
ommunication', 'Discrim*BF Communication', 'Discrim*PGM Communication'),table.margin = 0, center = TRUE, caption
= "The moderation of communication from allomothers (rows 3-5) on relationship of discrimination (row 2) on depre
ssion, state-anxiety, and perceived stress (columns 1-3, respectively), holding certain covariates constant (rows
5-10). Each cell contains the pooled beta, with stars indicating significance level and pooled robust standard er
rors in the parentheses. R2 and the total number of women in the study, are also presented.
Model comparison calculated from 5 imputed data sets against their respective null models produced the following
pooled (F-statistics; p-values): depression XX; <0.0001), state anxiety (XX ; <0.0001), perceived stress (XX; <0.
0001).
 DOUBLE CHECK F-STATS BEFORE SUBMITTING
")


#need a new way of finding Ftest for something with moderators...
```

Hide

```
output4
```

Hide

```
ggplot(df5,
       aes(x = CEEDS,
           y = CEPSS,
           color = MomCommBin)) +
  theme_bw() +
  labs(x = "Independent variable",
       y = "Dependent variable",
       color = "Moderator") +
  geom_point(alpha = .3,
             size = .9) +
  geom_smooth(method = "lm")

# Depression
d2_c <- ggplot(df2,
       aes(x = CEEDS,
           y = CEEPDS,
           color = MomCommBin)) +
  theme_bw() +
  labs(x = "Discrimination",
       y = "Depression",
       color = "Communication Levels with MGM") +
  geom_point(alpha = .3,
             size = .9) +
  geom_smooth(method = "lm")

#Anxiety

a2_c <- ggplot(df2,
       aes(x = CEEDS,
           y = CESTAISF,
           color = MomCommBin)) +
  theme_bw() +
  labs(x = "Discrimination",
       y = "Anxiety",
       color = "Communication Levels with MGM") +
  geom_point(alpha = .3,
             size = .9) +
  geom_smooth(method = "lm")
```

```
#Stress
s2_c <- ggplot(df2,
       aes(x = CEEDS,
           y = CEPSS,
           color = MomCommBin)) +
  theme_bw() +
  labs(x = "Discrimination",
       y = "Stress",
       color = "Communication Levels with MGM") +
  geom_point(alpha = .3,
             size = .9) +
  geom_smooth(method = "lm")

C <- ggarrange(d2_c, a2_c, s2_c + rremove("x.text"), common.legend = TRUE,
          labels = c("D", "E", "F"),
          ncol = 3, nrow = 1)

C
```

Supplementary Materials

Set 1 - Regression Diagnostics

Hide

```
supptab_Discnomod %>% gt() %>%
 tab_header(title = "Regression Diagnostics for Model 1")
```

Set 2 - Regression Diagnositics

Social Support

Hide

```
supptab_Disc_SocSuppMod_indiv %>% gt() %>%
 tab_header(title = "Regression Diagnostics for Model 2 Social Support")
```

Hide

```
vif(Stress_Disc_SocSuppMod_indiv[[X]])
```

Communication

Hide

```
supptab_Disc_CommMod_indiv %>% gt() %>%
 tab_header(title = "Regression Diagnostics for Model 2 Communication")
```

Geographic Proximity

Hide

```
supptab_Disc_GeoProxMod_indiv %>% gt() %>%
 tab_header(title = "Regression Diagnostics for Model 2 Geographic Proximity")
```

Post-hoc Analyses by Reviewer Request

Re-do Set 1 Stratified by Place of Birth

Hide

```
#POSSIBLE EXTRA -- SPLIT BY Language
```

Hide

```
mod_depression_discrimination_nomoderation_Forborn1 <- with(datlist, lm(CEEPDS ~ CEEDS +
                                                                  ses_full +
                                                                  age + tri+ Parity, subset = ForBorn == 1))

betas1 <- lapply( mod_depression_discrimination_nomoderation_Forborn1, FUN=function(rr){ coef(rr) } )
vars1 <- lapply( mod_depression_discrimination_nomoderation_Forborn1, FUN=function(rr){ vcov(rr) } ) #robust stan
dard errors
beta_names1 <- names(betas1[[1]])
cons1 <- beta_names1[-1]
summary(pool_mi(betas1, vars1))


mod1a <- extract.df(mod_depression_discrimination_nomoderation_Forborn1)
testEstimates(mod_depression_discrimination_nomoderation_Forborn1)
Ftest1a <- mitml::testConstraints( mod_depression_discrimination_nomoderation_Forborn1, constraints= cons1 )
print(Ftest1a)

mod_depression_discrimination_nomoderation_Forborn0 <- with(datlist, lm(CEEPDS ~ CEEDS +
                                                                  ses_full +
                                                                  age + tri+ Parity, subset = ForBorn == 0))

betas1 <- lapply( mod_depression_discrimination_nomoderation_Forborn0, FUN=function(rr){ coef(rr) } )
vars1 <- lapply( mod_depression_discrimination_nomoderation_Forborn0, FUN=function(rr){ vcov(rr) } ) #robust stan
dard errors
beta_names1 <- names(betas1[[1]])
cons1 <- beta_names1[-1]
summary(pool_mi(betas1, vars1))


mod1b <- extract.df(mod_depression_discrimination_nomoderation_Forborn0)
testEstimates(mod_depression_discrimination_nomoderation_Forborn0)
Ftest1b <- mitml::testConstraints( mod_depression_discrimination_nomoderation_Forborn0, constraints= cons1 )
print(Ftest1b)
```

Anxiety ~ Discrimination

Hide

```
# anxiety is not significant if adding other mental health variables....
anxiety_discrimination_nomoderation_Forborn1 <- with(imputed, lm(CESTAISF  ~ CEEDS +
                                                         I(normalize(SES) +
                                                           normalize(as.numeric(eduR)) +
normalize(as.numeric(foodsecure))) +
                                                         age + tri+ Parity, subset = ForBorn == 1))

summary(pool(anxiety_discrimination_nomoderation_Forborn1))
pool.r.squared(anxiety_discrimination_nomoderation_Forborn1) #not adjusted R^2


#anxiety
anxiety_discrimination_nomoderation_Forborn1 <- with(datlist, lm(CESTAISF ~ CEEDS +
                                                         ses_full +
                                                         age + tri+ Parity, subset = ForBorn == 1))

betas2 <- lapply( anxiety_discrimination_nomoderation_Forborn1, FUN=function(rr){ coef(rr) } )
vars2 <- lapply( anxiety_discrimination_nomoderation_Forborn1, FUN=function(rr){ vcov(rr) } ) #robust standard er
rors
beta_names2 <- names(betas2[[1]])
cons2 <- beta_names2[-1]
summary(pool_mi(betas2, vars2))


mod2a <- extract.df(anxiety_discrimination_nomoderation_Forborn1)
testEstimates(anxiety_discrimination_nomoderation_Forborn1)
Ftest2a <- mitml::testConstraints( anxiety_discrimination_nomoderation_Forborn1, constraints= cons2 )
print(Ftest2a)


###

mod_anxiety_discrimination_nomoderation_ForBorn0 <- with(imputed, lm(CESTAISF  ~ CEEDS +
                                                         I(normalize(SES) +
                                                           normalize(as.numeric(eduR)) +
normalize(as.numeric(foodsecure))) +
                                                         age + tri+ Parity, subset = ForBorn == 0))

summary(pool(mod_anxiety_discrimination_nomoderation_ForBorn0))
```

```
pool.r.squared(mod_anxiety_discrimination_nomoderation_ForBorn0) #not adjusted R^2


#anxiety
mod_anxiety_discrimination_nomoderation_ForBorn0 <- with(datlist, lm(CESTAISF ~ CEEDS +
                                                           ses_full +
                                                           age + tri+ Parity, subset = ForBorn == 0))

betas2 <- lapply( mod_anxiety_discrimination_nomoderation_ForBorn0, FUN=function(rr){ coef(rr) } )
vars2 <- lapply( mod_anxiety_discrimination_nomoderation_ForBorn0, FUN=function(rr){ vcov(rr) } ) #robust standar
d errors
beta_names2 <- names(betas2[[1]])
cons2 <- beta_names2[-1]
summary(pool_mi(betas2, vars2))


mod2b <- extract.df(mod_anxiety_discrimination_nomoderation_ForBorn0)
testEstimates(mod_anxiety_discrimination_nomoderation_ForBorn0)
Ftest2b <- mitml::testConstraints( mod_anxiety_discrimination_nomoderation_ForBorn0, constraints= cons2 )
print(Ftest2b)
```

Stress ~ Discrimination

Hide

```
# stress is significant
stress_discrimination_nomoderation_ForBorn1 <- with(imputed, lm( CEPSS~ CEEDS +
                                                         I(normalize(SES) +
                                                           normalize(as.numeric(eduR)) +
normalize(as.numeric(foodsecure))) +
                                                         age + tri+ Parity, subset = ForBorn == 1))

summary(pool(stress_discrimination_nomoderation_ForBorn1))
pool.r.squared(stress_discrimination_nomoderation_ForBorn1) #not adjusted R^2

stress_discrimination_nomoderation_ForBorn1 <- with(datlist, lm(CEPSS ~ CEEDS +
                                                         ses_full +
                                                         age + tri+ Parity, subset = ForBorn == 1 ))

betas3 <- lapply( stress_discrimination_nomoderation_ForBorn1, FUN=function(rr){ coef(rr) } )
vars3 <- lapply( stress_discrimination_nomoderation_ForBorn1, FUN=function(rr){ vcov(rr) } ) #robust standard err
ors
summary(pool_mi(betas3, vars3))
beta_names3 <- names(betas3[[1]])
cons3 <- beta_names3[-1]


mod3a <- extract.df(stress_discrimination_nomoderation_ForBorn1)
testEstimates(stress_discrimination_nomoderation_ForBorn1)
Ftest3a <- mitml::testConstraints( stress_discrimination_nomoderation_ForBorn1, constraints= cons3 )
print(Ftest3a)


#
stress_discrimination_nomoderation_ForBorn0 <- with(imputed, lm( CEPSS~ CEEDS +
                                                         I(normalize(SES) +
                                                           normalize(as.numeric(eduR)) +
normalize(as.numeric(foodsecure))) +
                                                         age + tri+ Parity, subset = ForBorn == 0))

summary(pool(stress_discrimination_nomoderation_ForBorn0))
pool.r.squared(stress_discrimination_nomoderation_ForBorn0) #not adjusted R^2

stress_discrimination_nomoderation_ForBorn0 <- with(datlist, lm(CEPSS ~ CEEDS +
```

```
                                                         ses_full +
                                                         age + tri+ Parity, subset = ForBorn == 0 ))

betas3 <- lapply( stress_discrimination_nomoderation_ForBorn0, FUN=function(rr){ coef(rr) } )
vars3 <- lapply( stress_discrimination_nomoderation_ForBorn0, FUN=function(rr){ vcov(rr) } ) #robust standard err
ors
summary(pool_mi(betas3, vars3))
beta_names3 <- names(betas3[[1]])
cons3 <- beta_names3[-1]


mod3b <- extract.df(stress_discrimination_nomoderation_ForBorn0)
testEstimates(stress_discrimination_nomoderation_ForBorn0)
Ftest3b <- mitml::testConstraints( stress_discrimination_nomoderation_ForBorn0, constraints= cons3 )
print(Ftest3b)
```

Hide

```
output <- texreg::htmlreg(list(mod1a,mod1b,mod2a,mod2b,mod3a,mod3b), digits = 2, custom.model.names = c('Depressi
on - FB','Depression - US', 'State Anxiety - FB','State Anxiety - US','Perceived Stress - FB','Perceived Stress -
US'), custom.gof.names = c('R-squared','N'),custom.coef.names = c('Intercept','Discrimination','Socio-Economic St
atus', 'Age','Trimester','Parity'),table.margin = 0, center = TRUE, caption = "The relationship of discrimination
(row 2) on depression, state-anxiety, and perceived stress (columns 1&2, 3&4, and 5&6, respectively), holding cer
tain covariates constant (rows 3-7). Each cell contains the pooled beta, with stars indicating significance level
and pooled robust standard errors in the parentheses. R2 and the total number of women in the study, are also pre
sented.
Model comparison calculated from 5 imputed data sets against their respective null models produced the following
pooled (F-statistics; p-values): depression 5.959; <0.0001), state anxiety (3.704; <0.0001), pregnancy-related an
xiety (5.314; <0.0001). DOUBLE CHECK F-STATS BEFORE SUBMITTING
")
```

Hide

```
output
```

Re-do Set 1 Stratified by Language (not imputed)

Hide

```
df_merged$Lang2 <- ifelse(df_merged$Lang == "Spanish",1,0)
mod <- lm(CEPSS ~ CEEDS + ses_full + age + I(normalize(SES) +
                                                   normalize(as.numeric(eduR)) +
                                              tri+ Parity, df_merged, subset = Lang2 == 1))

summary(mod)
mod <- lm(CEPSS ~ CEEDS + ses_full + age + I(normalize(SES) +
                                                   normalize(as.numeric(eduR)) +
                                              tri+ Parity, df_merged, subset = Lang2 == 0))
summary(mod)



# correlation matrices for reviewer
df_cor_slim <- dat %>% select(MomSocSupp,BFSocSupp,MILSocSupp,MomComm, BFComm, MILComm, MomGeoProx, BFGeoProx, MI
LGeoProx)

df_cor_slim <- dat %>% select(MomSocSupp,MomComm, MomGeoProx)
cormat <- round(cor(df_cor_slim, use = "complete.obs"),2)
cor_test_mat <- corr.test(df_cor_slim)$p    # Apply corr.test function
p.mat <- cor_pmat(df_cor_slim)
ggcorrplot(cormat, p.mat = p.mat, type = "lower",lab = TRUE)


df_cor_slim <- dat %>% select(BFSocSupp,BFComm, BFGeoProx)
cormat <- round(cor(df_cor_slim, use = "complete.obs"),2)
cor_test_mat <- corr.test(df_cor_slim)$p    # Apply corr.test function
p.mat <- cor_pmat(df_cor_slim)
ggcorrplot(cormat, p.mat = p.mat, type = "lower",lab = TRUE)

df_cor_slim <- dat %>% select(MILSocSupp,MILComm, MILGeoProx)
cormat <- round(cor(df_cor_slim, use = "complete.obs"),2)
cor_test_mat <- corr.test(df_cor_slim)$p    # Apply corr.test function
p.mat <- cor_pmat(df_cor_slim)
ggcorrplot(cormat, p.mat = p.mat, type = "lower",lab = TRUE)
```
