# Supplementary material for: Maternal grandmothers buffer the effects of ethnic discrimination among pregnant Latina mothers
Source: Evol Hum Sci. 2023 Nov 9;6:e7. doi: 10.1017/ehs.2023.27 (PMC10955360; doi:10.1017/ehs.2023.27)
Supplement: Knorr and Fox supplementary material 2 — Knorr and Fox supplementary material [file S2513843X23000270sup002.docx]

## **Supplemental Materials**

# **1.** **Operationalization of variables**

### 1.1 Everyday Discrimination Scale (EDS)

The EDS quantifies multiple aspects of ethnic/racial discrimination and the frequency of their occurrence with 9 items on a 4-point Likert scale. For the specific items and values see below.

**Supplemental Table 1**

| **EDS Item Text** | | | | |
| --- | --- | --- | --- | --- |
| **English** | | | **Spanish** | |
| Have you been treated with less respect than other people? | | | ¿Ha sido tratada con menos respeto que otras personas? | |
| Have you been treated unfairly at restaurants or stores? | | | ¿Ha sido tratada injustamente en restaurantes o tiendas? | |
| Have people criticized your accent or the way you speak? | | | ¿Ha sido criticada por su acento o la forma en la que habla? | |
| Have people acted as if they think you are not smart? | | | ¿Han habido personas que han actuado como si pensaran que usted no es inteligente? | |
| Have people acted as if they are afraid of you? | | | ¿Han habido personas que han actuado como si le tuvieran miedo? | |
| Have people acted as if they think you are dishonest? | | | ¿Han habido personas que han actuado como si pensaran que usted no es honesta? | |
| Have people acted as if they’re better than you are? | | | ¿Han habido personas que han actuado como si ellos fueran mejor que usted? | |
| Have you been threatened or harassed? | | | ¿Han habido personas que la han amenazado o acosado? | |
| Have you been followed around in stores? | | | ¿Ha sido monitoreada o seguida en tiendas departamentales? | |
| **EDS Item Values** | | | | |
| **Text of answer choice** | | | **Numeric value** |  |
| **English** | **Spanish** | |  |  |
| Never | Nunca | | 1 |  |
| Rarely | Casi nunca | | 2 |  |
| Sometimes | Algunas veces | | 3 |  |
| Often | Con frecuencia | | 4 |  |

### 1.2 Social Support

Social support was measured with the Multidimensional Scale of Perceived Social Support (MPSS) - Family Subscale (Zimet et al., 1988). From this validated scale, each item answer (“Not true”, “Somewhat true”, “Very true”) is scored 1-3, respectively, and an average score is taken. We modified this scale to measure social support from a particular individual, rather than family generally. We repeated this scale three times for baby’s father, maternal grandmother, and paternal grandmother. For the baby’s maternal grandmother, we used the term “my mother” (to denote the relationship to the participant) and for paternal grandmother we used “my baby’s father’s mother”.

For visualizations, we dichotomized the variable based on the combined median score of MGM and PGM social support levels. This was operationalized as “High” social support (greater than or equal to median score >= 2.36) or “Low” social support (less than the median score of 2.36).

### **Supplemental Table 2**: Item Breakdown of MPSS Scale

| **MPSS Scale Item Text** | | | | | |
| --- | --- | --- | --- | --- | --- |
| **English** | | **Spanish** | | | |
| My mother really tries to help me. | | | Mi madre realmente me trata de ayudar. | | |
| I get the emotional help and support I need from my mother. | | | Yo recibo el apoyo y ayuda emocional que necesito de mi madre. | | |
| I can talk about my problems with my mother. | | | Puedo hablar de mis problemas con mi madre. | | |
| My mother is willing to help me make decisions. | | | Mi madre dispuesta a ayudarme a tomar decisiones. | | |
| **MPSS Scale Item Values** | | | | | |
| **English** | **Spanish** | | | **Numeric Values** | |
| Not true | Nada cierto | | | | 1 |
| Somewhat true | Algo cierto | | | | 2 |
| Very true | Muy cierto | | | | 3 |

Since emotional and instrumental social support have been shown to exert impact on perinatal outcomes differently (Bedaso et al., 2021; Emmott & Mace, 2015), we ask if this validated, but generically named scale, reflects emotional or instrumental support. Item 2 and 3 of the MPSS clearly reflect emotional support, as they describe emotions and interpersonal interactions. Items 1 and 4 are less clear as they describe the assessment of willingness and intent. We explored if the items 1 and 4 act differently than items 2 and 3. With only the emotional support items (2 and 3), we found that the mean score only changed for 6 of the 216 women for the MGM score and 5 women for the PGM score compared to the original calculations for the overall MPSS scale. This along with the strong reliability of the scale (determined through high Cronbach alphas) suggests that items 1 and 4 are not adding noise to the emotional support questions. Therefore, we state that the MPSS scale overall reflects emotional support most strongly of the different forms of social support that can influence perinatal outcomes.

MCE Wave 1 was conducted with multiple versions of the survey in order to maximize the number of constructs assessed. Recruitment began April 15, 2016. We launched a new version (2.0) of the survey on June20, 2016, which added some survey scales not previously included. Out of the 310 women who completed surveys, 87 women received an early version without social support scales. We opted to drop these women to avoid non-random missingness (for more on this, see section 2.1 below).

### 1.2 Communication

Communication was assessed by a single question: “*How often do you communicate with [this person]*?” with response options 1 = *Every day*, 2 = *More than once a week*, 3 = *More than once a month*, 4 = *Once a month or less*, 5 = *Never*. We reverse-coded to ensure that greater numbers indicated greater levels of communication.

For visualizations, this was then operationalized as “High levels of communication” (once a week or more) or “Low levels of communication” (less than once a week).

### 1.3 Geographic Proximity

Geographic proximity was one question, “*How nearby does [this person] live*?”, with response options 1 = *In my home*, 2 = *In my neighborhood*, 3 = *Outside my neighborhood but close enough to visit during the day*, 4 *= Too far to visit during the day*. We reverse-coded this variable so greater numerical values were associated with greater levels of geographic proximity.

For visualizations, we created a binary variable operationalized as ‘Close’ (in the same home or neighborhood) or ‘Far’ (outside or further than the same neighborhood).

## 1.4 Mental Health

1.4.1 Depression

The Edinburgh Postnatal Depression Scale (EPDS; Cox et al., 1987; Murry & Cox, 1990) consists of 10 items measured on a 4-point scale. Each item was anchored on a 1–4-point scale (1 - “No, never” and 4 - “Yes, most of the time”).

### **Supplemental Table 3: Item Breakdown of EPDS Scale**

| **Reverse Coded** | **EPDS Scale Item Text** | |
| --- | --- | --- |
|  | **English** | **Spanish** |
|  | I have been able to laugh and see the funny side of things | He podido reír y ver el lado gracioso de las cosas |
|  | I have looked forward with enjoyment to things | He mirado al futuro con placer para hacer cosas |
| x | I have blamed myself unnecessarily when things went wrong | Me he culpado sin necesidad cuando las cosas marchaban mal |
|  | I have been anxious or worried for no good reason | He estado ansiosa y preocupada sin motivo |
| x | I have felt scared or panicky for no very good reason | He sentido miedo o pánico sin motivo alguno |
| x | Things have been getting on top of me | Las cosas me oprimen o agobian |
| x | I have been so unhappy that I have had difficulty sleeping | Me he sentido tan infeliz, que he tenido dificultad para dormir |
| x | I have felt sad or miserable | Me he sentido triste y desgraciada |
| x | I have been so unhappy that I have been crying | Me he sentido tan infeliz que he estado llorando |
| x | The thought of harming myself has occurred to me | He pensado en hacerme daño |

1.4.2 Anxiety

The Spielberger State-Trait Anxiety Inventory State scale (STAI; validated among pregnant women by Marteau & Bekker, 1992), consists of six items, three of which are reversed coded, and anchored on a 4-point scale (1 = Not at all to 4 = Very much) with items like, “*I am worried*;”

### **Supplemental Table 4 Item Breakdown of STAI-SF Scale**

| **Reverse Coded** | **STAI-SF Item Text** | |
| --- | --- | --- |
|  | **English** | **Spanish** |
| x | I feel calm | Me siento calmada |
|  | I feel upset | Me siento disgustada |
| x | I feel content | Me siento contenta |
| x | I am relaxed | Estoy relajada |
|  | I am worried | Estoy preocupada |
|  | I am tense | Estoy tensa |

1.4.3 Perceived Stress

The Perceived Stress Scale (PSS) is the most widely used psychological instrument for measuring the perception of stress. It measures the degree to which situations in one’s life are appraised as stressful. We have used the shortened version of the Perceived Stress Scale for the purpose of our study.

### **Supplemental Table 5 Item Breakdown of PSS Scale**

| **Reverse Coded** | **Item Text** | |
| --- | --- | --- |
|  | **English** | **Spanish** |
|  | How often have you felt that you were unable to control the important things in your life? | ¿Con qué frecuencia ha sentido que usted no puede controlar las cosas/momentos importantes en su vida? |
| x | How often have you felt confident about your ability to handle your personal problems? | ¿Con qué frecuencia se ha sentido usted segura acerca de su habilidad de manejar sus problemas personales? |
| x | How often have you felt that things were going your way? | ¿Con qué frecuencia ha sentido que las cosas van a su manera? |
|  | How often have you felt difficulties were piling up so high that you could not overcome them? | ¿Con qué frecuencia ha sentido que sus dificultades se acumulan tanto que no puede superarlas? |

As noted in the manuscript, we find the following reliabilities for depression ɑ=0.85(ɑE=0.86, ɑS=0.84); state-anxiety ɑ=0.81(ɑE=0.84, ɑS=0.77); and perceived stress ɑ=0.52(ɑE=0.86, ɑS=0.33). Of note is the low cronbach alpha for the perceived stress models. This low reliability seems to be driven by the Spanish survey takers. However, we make use of published translations of the perceived stress scale from Dr. Elysia Davis, as seen in (Davis et al., 2004, 2011).

For the post-hoc re-analysis of Set 1 model (which explores the relationship of discrimination and perceived stress), we stratified the model by survey language using the original unimputed dataset. We found the results to remain the same among English speakers (ß: 1.740, SE: 0.430, p-value: <0.001, 32 dropped for missingness, overall model F-statistic: 3.823 and p-value: 0.002) and rendered insignificant for the Spanish speakers (ß: 0.488, SE: 0.645, p-value: 0.453, 35 dropped for missingness, overall model F-statistic: 2.389 and p-value: 0.041). However, for both models the betas remained the same direction.

There are likely issues with the PSS scale, but it is beyond our capabilities to account for what these issues may be. While the low Cronbach alpha perhaps casts the stress results as less convincing, we feel that the high Cronbach alpha and similar patterns of anxiety and depression is evidence that these results are not spurious.

1.4.4 Mental Health Thresholds

The clinically significant cut-off thresholds presented in the main paper were only included in Table 1 to help describe the cohort; these thresholds were calculated the following ways:

For the Edinburgh Perinatal Depression Scale (Cox et al., 1987, 1996; Santos et al., 2007), scores were calculated based on a summation of individual items. These summary scores can range from 0-30. Scores greater than 10 were identified as likely for at least minor depression. While there is some debate over the proper cut-off score to use, a validation of the EPDS among Spanish-speaking women suggests a cut-off threshold of 10 or 11 (Garcia-Esteve et al., 2003).

# 1.5 Control Variables

While the methods used in this paper do not allow for causal analysis, the relationship between discrimination and psychological distress has been previously established as causal (Williams et al., 2018). Therefore, we can make use of directed acyclic graphs to help construct our model (Pearl, 1995). A simplified DAG presented here shows that there are many overlapping factors involved in the high comorbidity of psychological distress measures. Each outcome variable represents a collider variable where there are backdoor paths. A factor of collider variables is that backdoor paths are already blocked, so controlling for these variables would open these paths and create possible confounding. Therefore, we do not control for mental health variables in the discrimination to psychological distress regression models and continue the same model design in Set 2 (where interaction terms are added).

**Supplemental Figure 1:**

# ~~
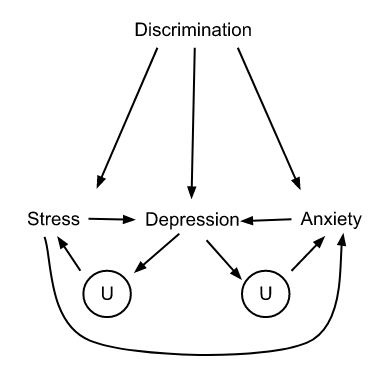
~~

**Supplemental Figure 1 Caption**: Directed Acyclic Graph (DAG) shows the relationship of variables in our first model (discrimination on mental health). Many arrows are shared between the mental health variables. We do not control for other mental health measures in our models in order to not open backdoor paths.

**Supplemental Figure 2:**


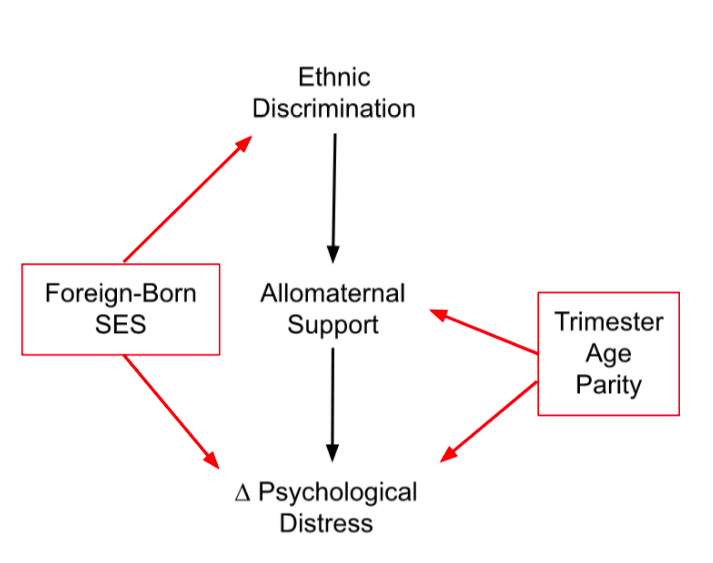


**Supplemental Figure 2 Caption**: Set 2 measures allomaternal support as a moderator of the relationship of ethnic discrimination and psychological distress, represented in DAG format as change in psychological distress. This graph shows how potential confounders (foreign-born, socioeconomic status (SES), trimester, age, parity) relate to our proposed model. While our model is based on observational data and cannot be interpreted causally, this conceptual modeling offers insights into how we surmise the variables are related.

# **2. Missingness and Imputation**

We examined missingness through the Amelia package. Overall, 7% of the observations were left blank. Individual variables ranged in missing from 1.4% (foreign-born) to 12.0% (food insecurity).

Multiple chain imputation was conducted on the 216 individuals using additional highly correlated variables not in the main analysis to improve accuracy of imputations. Such correlated variables used for imputation analysis included questions on how often you see your baby’s grandmother or father in person, general measures of overall family social support, age of immigration, as well as the acculturation level of the pregnant woman, which has been previously shown to be associated with levels of ethnic discrimination (Fox, 2021). Subject IDs were not included in imputation. We conducted 20 iterations of imputation for each of the five imputed datasets using the MICE package.

Density plots were used to determine if imputation was within range for all variables and if the distributions of each variable was maintained across imputed datasets. Red lines indicate the distribution of imputed variables, while blue lines are the distribution of the real data (Figure S3).

### **Supplemental Figure 3**: Density Diagnostic Plot of Imputed Variables


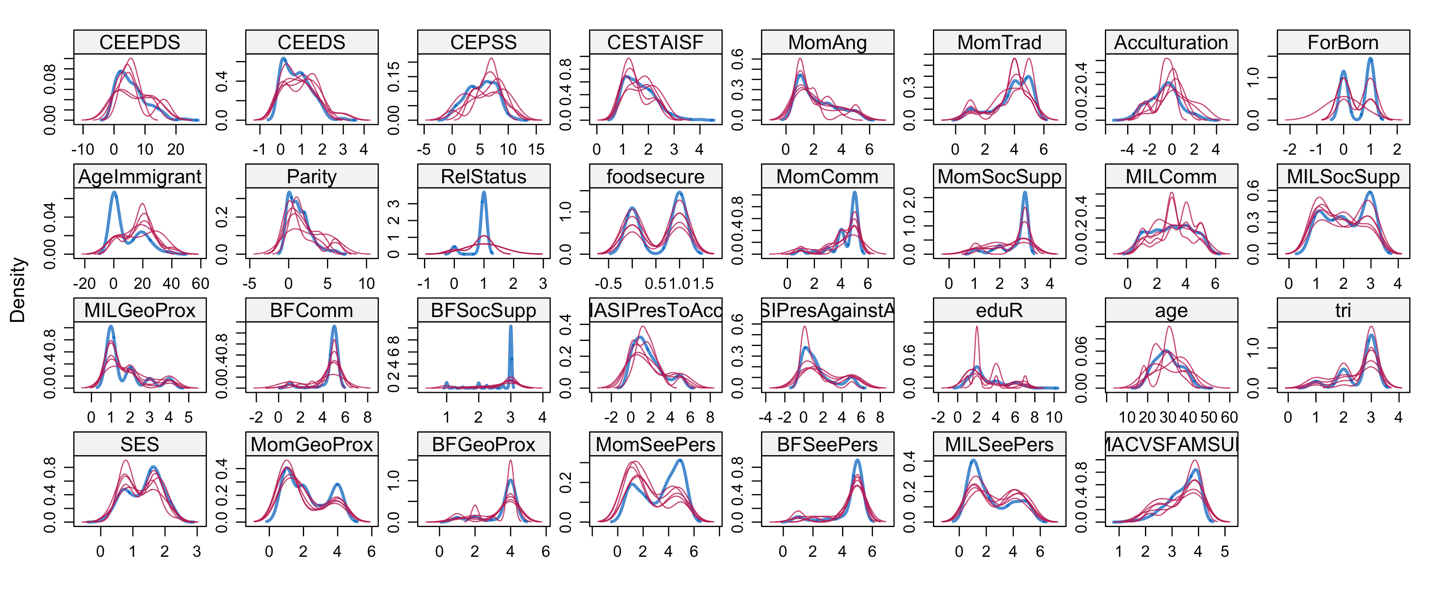


**2.1 Missing at Random Check**

The participants who received Version 1.0 of the survey and therefore missing social support scales were not included in the analytic dataset and therefore not relevant for determining missingness at random (for those, it’s as if that was an entirely separate study). Since no other systemic missingness is expected, the only other potential cause of nonrandom missingness in the remaining dataset would be participant characteristics. We considered the most likely participant characteristics to cause non-random missingness to be depression status and foreign-born status. We conducted t-tests to explore this question and found that missingness is not statistically different between depressed and non-depressed participants (t-statistic: 1.70, df: 41.35, p-value: 0.10). Missingness was also not statistically different between foreign-born and U.S.-born Latinos (t-statistic: 1.96, df: 209.03, p-value = 0.051); however, it closely approached the commonly used significance threshold of p-value <0.05. To explore this further, we post-hoc stratified the Set 1 models by birthplace.

Overall, we find that discrimination remains significant and positively associated with all psychological distress measures for both foreign-born and U.S. born women (see Supplemental Table 6). Thus, we determine that it is likely that our findings are not driven by any asymmetrical missingness.

**Supplemental Table 6: Set 1 Models Stratified by Place of Birth**

|  | Depression - FB | Depression - US | State Anxiety - FB | State Anxiety - US | Perceived Stress - FB | Perceived Stress - US |
| --- | --- | --- | --- | --- | --- | --- |
| Intercept | 4.19 | 5.48 | 1.31*** | 2.04*** | 2.88 | 5.44** |
|  | (3.08) | (3.31) | (0.33) | (0.42) | (1.56) | (1.85) |
| Discrimination | 2.45*** | 2.83*** | 0.17* | 0.26** | 1.46*** | 1.09* |
|  | (0.71) | (0.71) | (0.08) | (0.10) | (0.37) | (0.42) |
| Socio-Economic Status | -1.07 | -0.80 | -0.08 | -0.10 | -0.51 | -0.33 |
|  | (0.63) | (0.63) | (0.07) | (0.09) | (0.29) | (0.38) |
| Age | 0.07 | -0.09 | 0.01 | -0.01 | 0.04 | -0.05 |
|  | (0.08) | (0.11) | (0.01) | (0.01) | (0.04) | (0.07) |
| Trimester | -0.17 | 0.67 | -0.02 | -0.03 | 0.30 | 0.45 |
|  | (0.91) | (0.80) | (0.09) | (0.10) | (0.45) | (0.43) |
| Parity | -0.29 | -0.18 | -0.00 | -0.03 | -0.07 | -0.34 |
|  | (0.37) | (0.41) | (0.04) | (0.05) | (0.18) | (0.22) |
| R-squared | 0.18 | 0.19 | 0.10 | 0.12 | 0.22 | 0.14 |
| N | 120 | 96 | 120 | 96 | 120 | 96 |

**Supplemental Table 6 caption:** This table parallels Table 2 in the manuscript, and only differs by being stratified on Foreign Born (FB) vs. U.S.-born (US). The new sample size for these models are 120 and 96, respectively. Each cell contains the pooled beta, with stars indicating significance level and pooled robust standard errors in the parentheses. R^2^, the pooled coefficient of determination indicating how much variation in mental health is explained by the predictor and control variables and N (or total women in the study) are also presented. Model comparison calculated from 5 imputed data sets against their respective null models produced the following pooled (FB/US: F-statistics; p-values): depression (FB: 3.693; 0.003; US: 3.365; 0.005) state anxiety (FB: 2.185; 0.053; US: 2.153; 0.057), perceived stress (FB: 5.223; <0.001; US: 2.456; 0.032).

# **3.** **Regression Diagnostics**

For all models, we conducted Breusch-Pagan tests, which tests the null hypothesis of homoskedasticity. We failed to reject the null hypothesis in all state anxiety models and most perceived stress models in both Set 1 and Set 2. Therefore, we used robust standard errors in all models to account for heteroskedasticity and remain conservative in our estimates.

Variance-inflation factors (VIF) were calculated to examine correlations between independent variables to quantify multicollinearity. While some scholars suggest a VIF of 5 or 10 to be problematic (Menard, 2002), others suggest a more conservative 2.5 limit (Johnston et al., 2018). For Set 1, no VIF score was above 2.0. Given the interaction terms added in Set 2, VIF calculations are not appropriate (McClelland et al., 2017). However, given the lack of multicollinearity in Set 1 and the lack of multicollinearity in allomother characteristics with maternal mental health in our previous study (Knorr & Fox, 2023), we do not expect this to be an issue. A breakdown of these calculations can be found in Table S7-S9.

**Supplemental Table 7:**

| Regression Diagnostics for Set 1 Models | | | |
| --- | --- | --- | --- |
|  | | | |
| Model | Maximum VIF | Breusch Pagan p-value | Adjusted R-squared |
| Depression Imputation #1 | 1.28 | 0.012 | 0.16 |
| Depression Imputation #2 | 1.26 | 0.039 | 0.16 |
| Depression Imputation #3 | 1.24 | 0.02 | 0.14 |
| Depression Imputation #4 | 1.26 | 0.073 | 0.15 |
| Depression Imputation #5 | 1.26 | 0.058 | 0.14 |
| State Anxiety Imputation #1 | 1.65 | 0.447 | 0.43 |
| State Anxiety Imputation #2 | 1.63 | 0.591 | 0.45 |
| State Anxiety Imputation #3 | 1.61 | 0.25 | 0.44 |
| State Anxiety Imputation #4 | 1.63 | 0.493 | 0.44 |
| State Anxiety Imputation #5 | 1.6 | 0.561 | 0.44 |
| Perceived Stress Imputation #1 | 1.97 | 0.28 | 0.36 |
| Perceived Stress Imputation #2 | 2.04 | 0.372 | 0.36 |
| Perceived Stress Imputation #3 | 1.93 | 0.096 | 0.35 |
| Perceived Stress Imputation #4 | 1.97 | 0.163 | 0.35 |
| Perceived Stress Imputation #5 | 1.92 | 0.347 | 0.35 |

Set 2 includes the addition of interaction terms. Since VIF calculations are not appropriate for interaction models (McClelland et al., 2017), we do not include that column here, but otherwise reproduce the calculations from the previous supplemental table. Similar to Set 1 some tests for heteroskedasticity fail to reject the null, so we use robust standard errors for all models. Column 4 is the adjusted R^2^ for each model, which was calculated in order to describe how much variance of the outcome variable is associated with the predictors, including a penalty to account for the number of predictor variables. Residual plots run iteratively on each model for each imputed dataset did not reveal anything out of the ordinary (e.g., no non-linear relationships or high leverage data points).

**Supplemental Table 8:**

| Regression Diagnostics for Set 2 - Social Support | | |
| --- | --- | --- |
|  | | |
| Model | Breusch Pagan p-value | Adjusted R-squared |
| Depression Imputation #1 | 0 | 0.26 |
| Depression Imputation #2 | 0 | 0.26 |
| Depression Imputation #3 | 0 | 0.24 |
| Depression Imputation #4 | 0 | 0.27 |
| Depression Imputation #5 | 0 | 0.23 |
| State Anxiety Imputation #1 | 0.059 | 0.15 |
| State Anxiety Imputation #2 | 0.037 | 0.15 |
| State Anxiety Imputation #3 | 0.043 | 0.15 |
| State Anxiety Imputation #4 | 0.059 | 0.16 |
| State Anxiety Imputation #5 | 0.029 | 0.17 |
| Perceived Stress Imputation #1 | 0.283 | 0.19 |
| Perceived Stress Imputation #2 | 0.535 | 0.19 |
| Perceived Stress Imputation #3 | 0.371 | 0.15 |
| Perceived Stress Imputation #4 | 0.205 | 0.16 |
| Perceived Stress Imputation #5 | 0.442 | 0.17 |

**Supplemental Table 9:**

| Regression Diagnostics for Set 2 - Communication | | |
| --- | --- | --- |
|  | | |
| Model | Breusch Pagan p-value | Adjusted R-squared |
| Depression Imputation #1 | 0.016 | 0.22 |
| Depression Imputation #2 | 0.114 | 0.2 |
| Depression Imputation #3 | 0.081 | 0.19 |
| Depression Imputation #4 | 0.036 | 0.21 |
| Depression Imputation #5 | 0.063 | 0.19 |
| State Anxiety Imputation #1 | 0.206 | 0.1 |
| State Anxiety Imputation #2 | 0.075 | 0.1 |
| State Anxiety Imputation #3 | 0.052 | 0.12 |
| State Anxiety Imputation #4 | 0.183 | 0.11 |
| State Anxiety Imputation #5 | 0.099 | 0.12 |
| Perceived Stress Imputation #1 | 0.44 | 0.17 |
| Perceived Stress Imputation #2 | 0.436 | 0.16 |
| Perceived Stress Imputation #3 | 0.663 | 0.13 |
| Perceived Stress Imputation #4 | 0.503 | 0.14 |
| Perceived Stress Imputation #5 | 0.357 | 0.14 |

**Supplemental Table 10:**

| Regression Diagnostics for Set 2 - Geographic Proximity | | |
| --- | --- | --- |
|  | | |
| Model | Breusch Pagan p-value | Adjusted R-squared |
| Depression Imputation #1 | 0.074 | 0.16 |
| Depression Imputation #2 | 0.049 | 0.15 |
| Depression Imputation #3 | 0.11 | 0.13 |
| Depression Imputation #4 | 0.183 | 0.14 |
| Depression Imputation #5 | 0.112 | 0.11 |
| State Anxiety Imputation #1 | 0.141 | 0.1 |
| State Anxiety Imputation #2 | 0.001 | 0.08 |
| State Anxiety Imputation #3 | 0.041 | 0.1 |
| State Anxiety Imputation #4 | 0.005 | 0.09 |
| State Anxiety Imputation #5 | 0.002 | 0.08 |
| Perceived Stress Imputation #1 | 0.148 | 0.14 |
| Perceived Stress Imputation #2 | 0.016 | 0.14 |
| Perceived Stress Imputation #3 | 0.142 | 0.11 |
| Perceived Stress Imputation #4 | 0.025 | 0.11 |
| Perceived Stress Imputation #5 | 0.109 | 0.1 |

**Supplemental Table 11**:

Demographics of the study cohort, including both descriptive statistics of the measures in this study and those not in the study but relevant to understanding the cohort.

|  | **Total (N=216)** |
| --- | --- |
| **Where does your mother (baby’s maternal grandmother) currently live?** |  |
| U.S. | 134 (62.0%) |
| Mexico | 48 (22.2%) |
| Other | 15 (6.9%) |
| Missing | 19 (8.8%) |
| **Where does your baby's paternal grandmother currently live?** |  |
| U.S. | 94 (43.5%) |
| Mexico | 72 (33.3%) |
| Another country | 23 (10.6%) |
| Missing | 27 (12.5%) |
| **Where does your baby's father currently live?** |  |
| U.S. | 189 (87.5%) |
| Mexico | 12 (5.6%) |
| Another country | 5 (2.3%) |
| Missing | 10 (4.6%) |
| **Communication Levels with baby's MGM** |  |
| Talks once a week or more | 171 (79.2%) |
| Talks less than once a week | 29 (13.4%) |
| Missing | 16 (7.4%) |
| **Communication Levels with baby's PGM** |  |
| Talks once a week or more | 76 (35.2%) |
| Talks less than once a week | 113 (52.3%) |
| Missing | 27 (12.5%) |
| **Communication Levels with baby's father** |  |
| Talks once a week or more | 189 (87.5%) |
| Talks less than once a week | 17 (7.9%) |
| Missing | 10 (4.6%) |
| **Geographic Proximity to baby's MGM** |  |
| Lives in the same home or neighborhood | 120 (55.6%) |
| Lives in different neighborhood | 74 (34.3%) |
| Missing | 22 (10.2%) |
| **Geographic Proximity to baby's PGM** |  |
| Lives in the same home or neighborhood | 74 (34.3%) |
| Lives in different neighborhood | 114 (52.8%) |
| Missing | 28 (13.0%) |
| **Geographic Proximity to baby's father** |  |
| Lives in the same home or neighborhood | 184 (85.2%) |
| Lives in different neighborhood | 17 (7.9%) |
| Missing | 15 (6.9%) |
| **Depression (clinically significant symptoms)** |  |
| Depressed (>10) | 36 (16.7%) |
| Not Depressed | 172 (79.6%) |
| Missing | 8 (3.7%) |
| **State Anxiety (clinically significant symptoms)** |  |
| Anxious (>2) | 49 (22.7%) |
| Not Anxious | 156 (72.2%) |
| Missing | 11 (5.1%) |

Within each allomothers’ relationship with the mother, each relationship characteristic (social support, communication, and geographic proximity) is significantly and positively correlated with the other relationship characteristics. It is important to note that these relationship characteristics are not perfectly correlated with each other, suggesting that these variables overlap in the phenomena they capture but they do not represent a unified factor.

MGM:

**
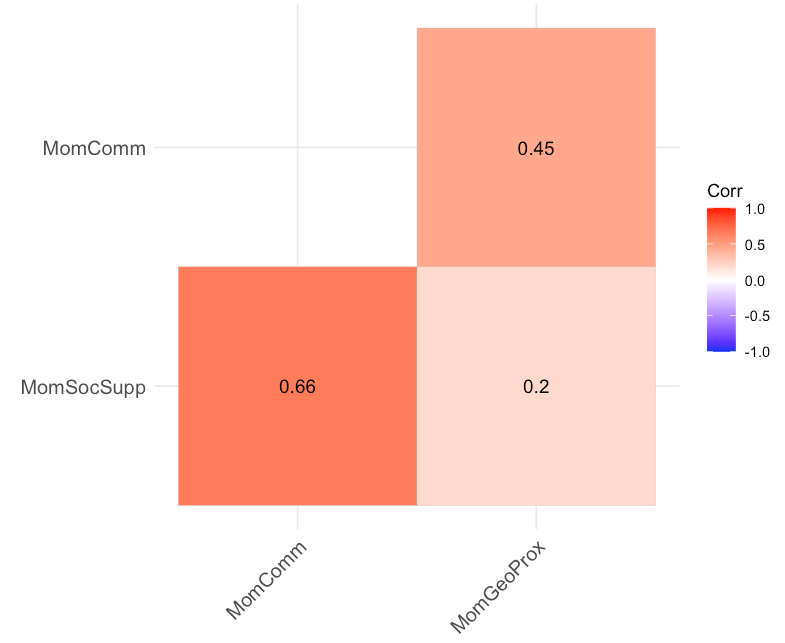
**

PGM:

**
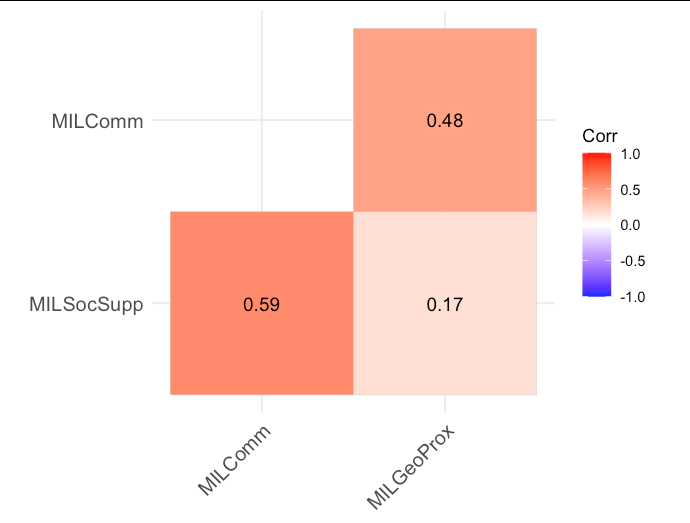
**

Father:


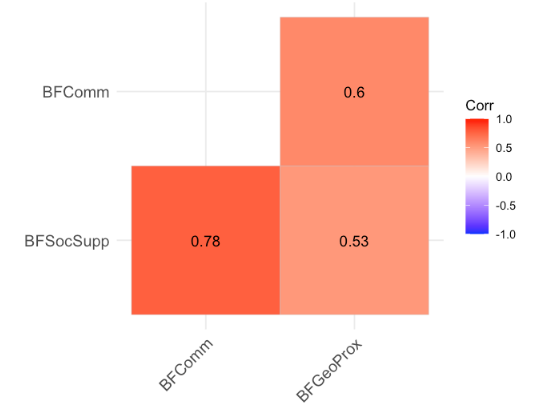


**References**

Bedaso, A., Adams, J., Peng, W., & Sibbritt, D. (2021). The relationship between social support and mental health problems during pregnancy: A systematic review and meta-analysis. *Reproductive Health*, *18*(1), 162. https://doi.org/10.1186/s12978-021-01209-5

Cox, J. L., Chapman, G., Murray, D., & Jones, P. (1996). Validation of the Edinburgh Postnatal Depression Scale (EPDS) in non-postnatal women. *Journal of Affective Disorders*, *39*(3). https://doi.org/10.1016/0165-0327(96)00008-0

Cox, J. L., Holden, J. M., & Sagovsky, R. (1987). Detection of postnatal depression. Development of the 10-item Edinburgh Postnatal Depression Scale. *The British Journal of Psychiatry: The Journal of Mental Science*, *150*, 782–786. https://doi.org/10.1192/bjp.150.6.782

Davis, E. P., Glynn, L. M., Waffarn, F., & Sandman, C. A. (2011). Prenatal maternal stress programs infant stress regulation. *Journal of Child Psychology and Psychiatry, and Allied Disciplines*, *52*(2), 119–129. https://doi.org/10.1111/j.1469-7610.2010.02314.x

Davis, E. P., Snidman, N., Wadhwa, P. D., Glynn, L. M., Schetter, C. D., & Sandman, C. A. (2004). Prenatal Maternal Anxiety and Depression Predict Negative Behavioral Reactivity in Infancy. *Infancy*, *6*(3), 319–331. https://doi.org/10.1207/s15327078in0603_1

Emmott, E. H., & Mace, R. (2015). Practical Support from Fathers and Grandmothers Is Associated with Lower Levels of Breastfeeding in the UK Millennium Cohort Study. *PLOS ONE*, *10*(7), e0133547. https://doi.org/10.1371/journal.pone.0133547

Fox, M. (2021). Discrimination as a Moderator of the Effects of Acculturation and Cultural Values on Mental Health Among Pregnant and Postpartum Latina Women. *American Anthropologist*, *123*(4), 780–804. https://doi.org/10.1111/aman.13665

Garcia-Esteve, L., Ascaso, C., Ojuel, J., & Navarro, P. (2003). Validation of the Edinburgh Postnatal Depression Scale (EPDS) in Spanish mothers. *Journal of Affective Disorders*, *75*(1), 71–76. https://doi.org/10.1016/s0165-0327(02)00020-4

Johnston, R., Jones, K., & Manley, D. (2018). Confounding and collinearity in regression analysis: A cautionary tale and an alternative procedure, illustrated by studies of British voting behaviour. *Quality & Quantity*, *52*(4), 1957–1976. https://doi.org/10.1007/s11135-017-0584-6

Knorr, D. A., & Fox, M. (2023). An evolutionary perspective on the association between grandmother-mother relationships and maternal mental health among a cohort of pregnant Latina women. *Evolution and Human Behavior*, *44*(1), 30–38. https://doi.org/10.1016/j.evolhumbehav.2022.10.005

McClelland, G. H., Irwin, J. R., Disatnik, D., & Sivan, L. (2017). Multicollinearity is a red herring in the search for moderator variables: A guide to interpreting moderated multiple regression models and a critique of Iacobucci, Schneider, Popovich, and Bakamitsos (2016). *Behavior Research Methods*, *49*(1), 394–402. https://doi.org/10.3758/s13428-016-0785-2

Menard, S. (2002). *Applied Logistic Regression Analysis*. SAGE Publications, Inc. https://doi.org/10.4135/9781412983433

Pearl, J. (1995). Causal diagrams for empirical research. *Biometrika*, *82*(4), 669–688. https://doi.org/10.1093/biomet/82.4.669

Santos, I. S., Matijasevich, A., Tavares, B. F., Barros, A. J. D., Botelho, I. P., Lapolli, C., Magalhães, P. V. da S., Barbosa, A. P. P. N., & Barros, F. C. (2007). Validation of the Edinburgh Postnatal Depression Scale (EPDS) in a sample of mothers from the 2004 Pelotas Birth Cohort Study. *Cadernos de Saúde Pública*, *23*(11), 2577–2588. https://doi.org/10.1590/S0102-311X2007001100005

Williams, M. T., Printz, D. M. B., & DeLapp, R. C. T. (2018). Assessing racial trauma with the Trauma Symptoms of Discrimination Scale. *Psychology of Violence*, *8*(6), 735–747. https://doi.org/10.1037/vio0000212

Zimet, G. D., Dahlem, N. W., Zimet, S. G., & Farley, G. K. (1988). The Multidimensional Scale of Perceived Social Support. *Journal of Personality Assessment*, *52*(1), 30–41. https://doi.org/10.1207/s15327752jpa5201_2
