# Supplementary figures and images for: Maternal grandmothers buffer the effects of ethnic discrimination among pregnant Latina mothers
Source: Evol Hum Sci. 2023 Nov 9;6:e7. doi: 10.1017/ehs.2023.27 (PMC10955360; doi:10.1017/ehs.2023.27)

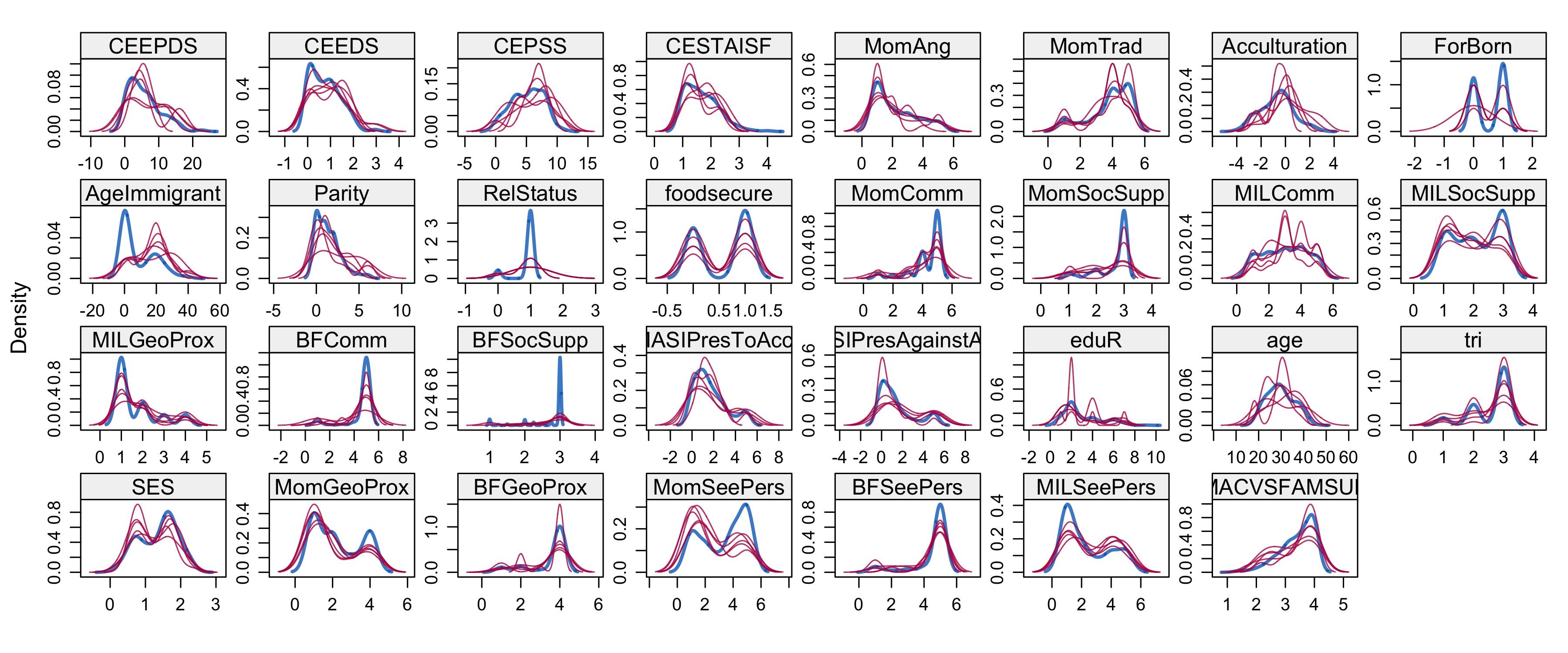

Supplement: Knorr and Fox supplementary material 3 — Knorr and Fox supplementary material [file S2513843X23000270sup003.zip › SuppFig3.tif]

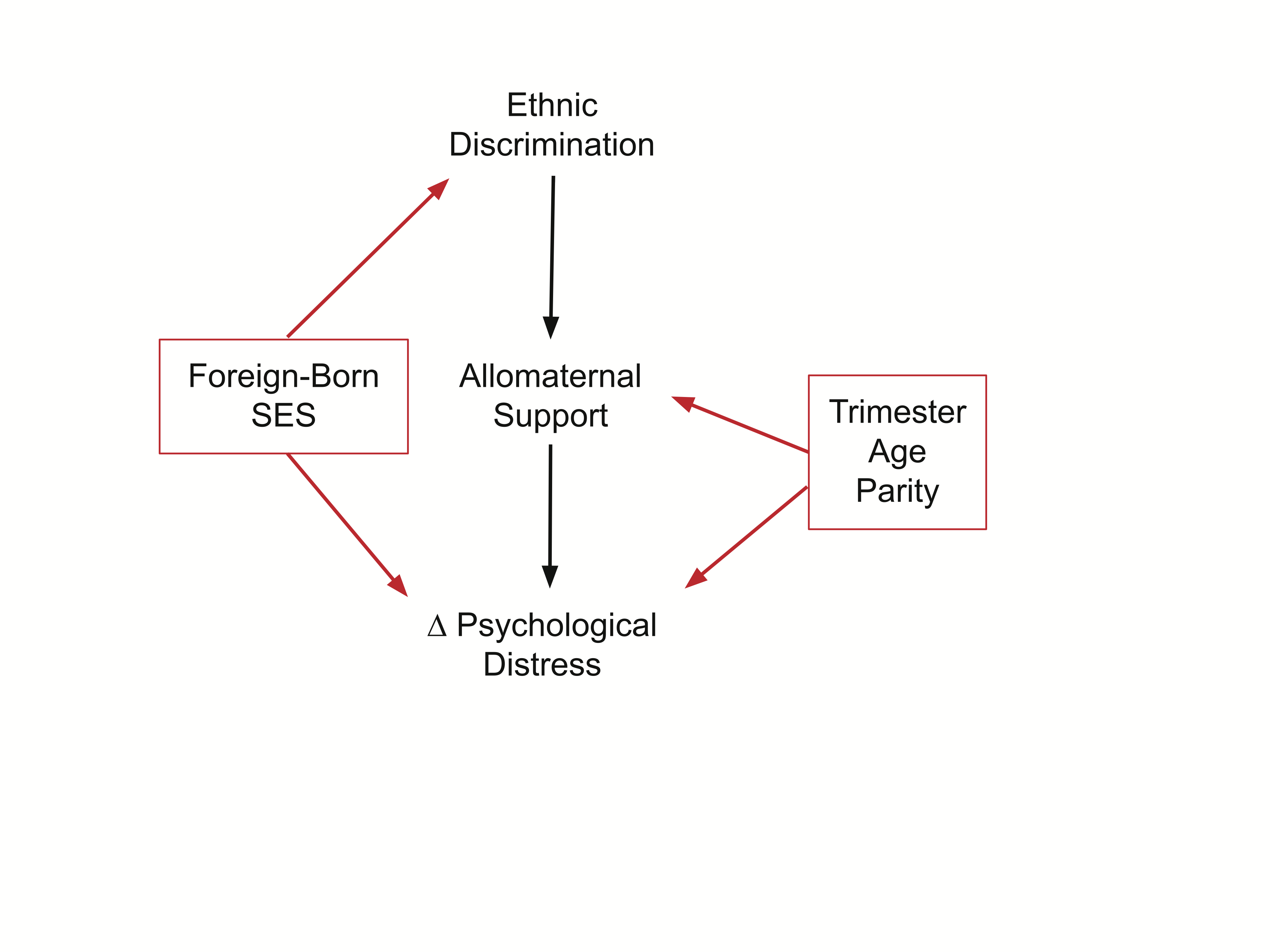

Supplement: Knorr and Fox supplementary material 3 — Knorr and Fox supplementary material [file S2513843X23000270sup003.zip › SuppFig2.tiff]

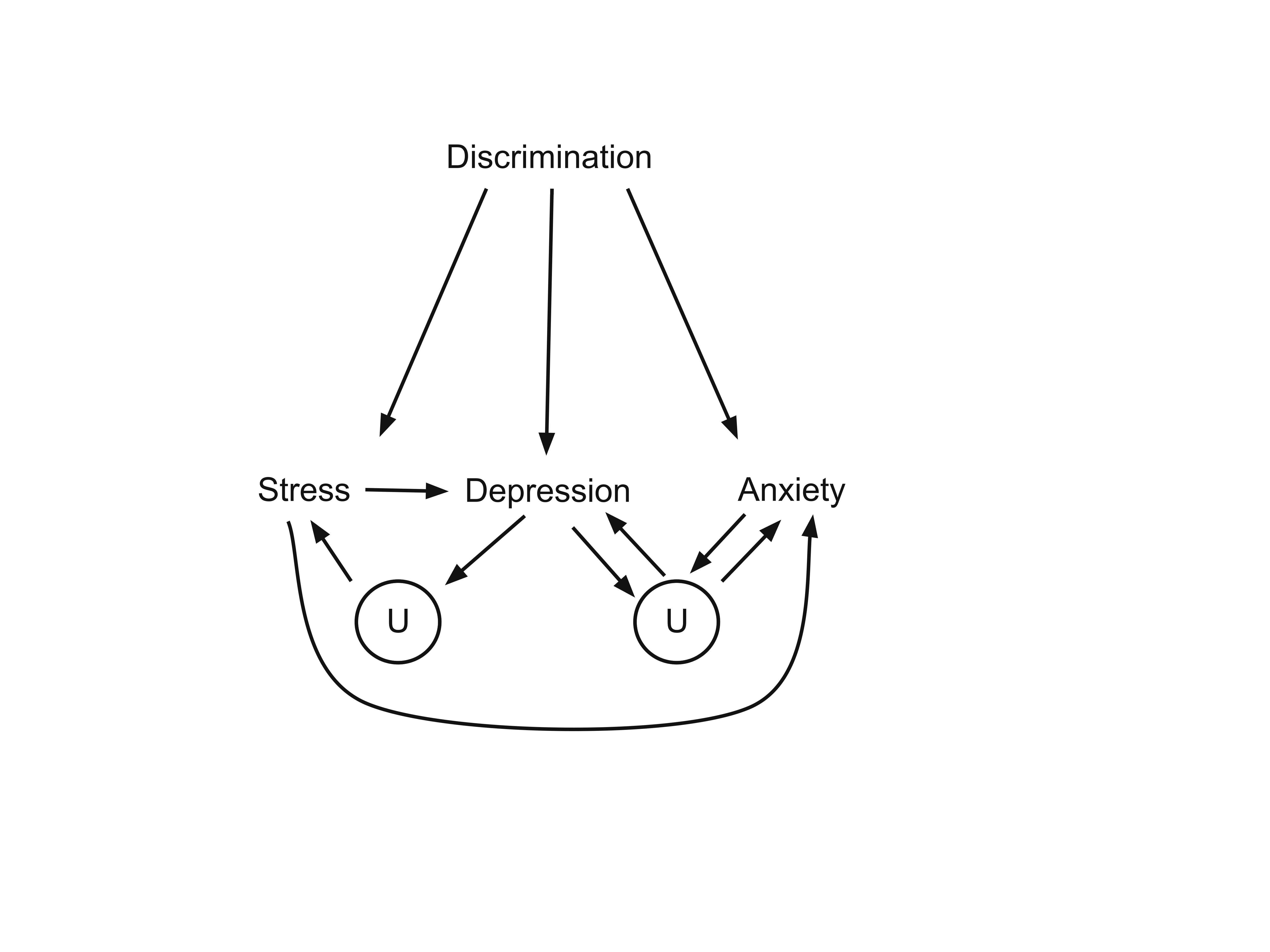

Supplement: Knorr and Fox supplementary material 3 — Knorr and Fox supplementary material [file S2513843X23000270sup003.zip › SuppFig1.tiff]

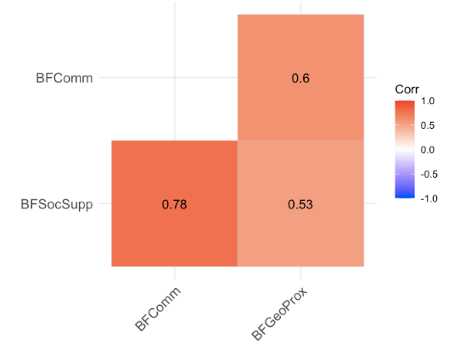

Supplement: Knorr and Fox supplementary material 3 — Knorr and Fox supplementary material [file S2513843X23000270sup003.zip › SM_Fig4c.tiff]

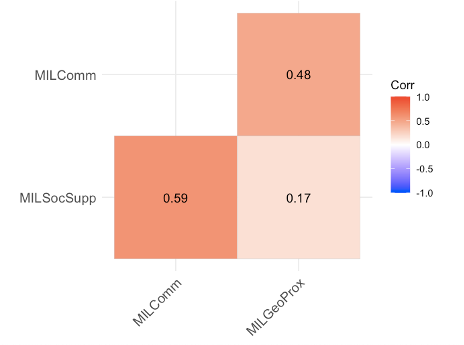

Supplement: Knorr and Fox supplementary material 3 — Knorr and Fox supplementary material [file S2513843X23000270sup003.zip › SM_Fig4b.tiff]

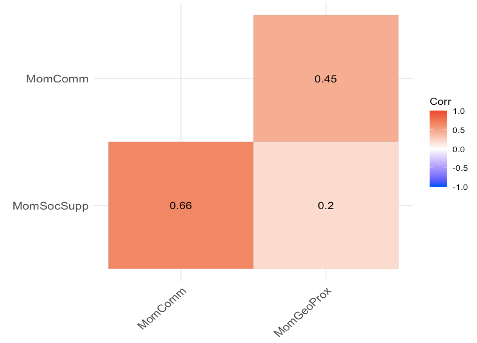

Supplement: Knorr and Fox supplementary material 3 — Knorr and Fox supplementary material [file S2513843X23000270sup003.zip › SM_Fig4a.png]
